# An adaptive bin framework search method for a beta-sheet protein homopolymer model

**DOI:** 10.1186/1471-2105-8-136

**Published:** 2007-04-24

**Authors:** Alena Shmygelska, Holger H Hoos

**Affiliations:** 1 Department of Structural Biology, Stanford University, 299 W. Campus Dr., Stanford, CA 94305, USA; 2 Department of Computer Science, University of British Columbia, 2366 Main Mall, Vancouver, BC V6T 1Z4, Canada

## Abstract

**Background:**

The problem of protein structure prediction consists of predicting the functional or *native *structure of a protein given its linear sequence of amino acids. This problem has played a prominent role in the fields of biomolecular physics and algorithm design for over 50 years. Additionally, its importance increases continually as a result of an exponential growth over time in the number of known protein sequences in contrast to a linear increase in the number of determined structures. Our work focuses on the problem of searching an exponentially large space of possible conformations as efficiently as possible, with the goal of finding a global optimum with respect to a given energy function. This problem plays an important role in the analysis of systems with complex search landscapes, and particularly in the context of *ab initio *protein structure prediction.

**Results:**

In this work, we introduce a novel approach for solving this conformation search problem based on the use of a bin framework for adaptively storing and retrieving promising locally optimal solutions. Our approach provides a rich and general framework within which a broad range of adaptive or reactive search strategies can be realized. Here, we introduce adaptive mechanisms for choosing which conformations should be stored, based on the set of conformations already stored in memory, and for biasing choices when retrieving conformations from memory in order to overcome search stagnation.

**Conclusion:**

We show that our bin framework combined with a widely used optimization method, Monte Carlo search, achieves significantly better performance than state-of-the-art generalized ensemble methods for a well-known protein-like homopolymer model on the face-centered cubic lattice.

## Background

Considering the close connection between the function of proteins and their three-dimensional (tertiary) structure, there are many reasons for studying protein folding; these include the desire to predict protein function based on sequence data (*via *tertiary structure prediction), to better understand a number of diseases that are directly caused by protein misfolding, aggregation and fibrillogenesis (some of which include Alzheimer's, Huntington's and prion disease as well as cystic fibrosis [1]), and to design proteins with desired structure and function. Since experimental methods (X-ray crystallography and NMR) for protein structure determination are highly labour intensive and require purification and, in the case of X-ray crystallography, crystallization of proteins, computational methods for predicting protein structure from sequence are very attractive.

The *ab initio *protein folding problem is the problem of predicting the tertiary structure (the *native *state) of a protein from its amino acid sequence by minimizing a given energy function. Even for simple models that discretize conformations on a lattice (grid), this optimization problem is N
 MathType@MTEF@5@5@+=feaafiart1ev1aaatCvAUfKttLearuWrP9MDH5MBPbIqV92AaeXatLxBI9gBamrtHrhAL1wy0L2yHvtyaeHbnfgDOvwBHrxAJfwnaebbnrfifHhDYfgasaacH8akY=wiFfYdH8Gipec8Eeeu0xXdbba9frFj0=OqFfea0dXdd9vqai=hGuQ8kuc9pgc9s8qqaq=dirpe0xb9q8qiLsFr0=vr0=vr0dc8meaabaqaciaacaGaaeqabaWaaeGaeaaakeaaimaacqWFneVtaaa@383B@*P*-hard [2,3]. Its difficulty stems from the fact that the space of possible conformations is vast, and the search landscapes induced by the given energy function are very complex. One of the most prominent and successful approaches for solving this and many other N
 MathType@MTEF@5@5@+=feaafiart1ev1aaatCvAUfKttLearuWrP9MDH5MBPbIqV92AaeXatLxBI9gBamrtHrhAL1wy0L2yHvtyaeHbnfgDOvwBHrxAJfwnaebbnrfifHhDYfgasaacH8akY=wiFfYdH8Gipec8Eeeu0xXdbba9frFj0=OqFfea0dXdd9vqai=hGuQ8kuc9pgc9s8qqaq=dirpe0xb9q8qiLsFr0=vr0=vr0dc8meaabaqaciaacaGaaeqabaWaaeGaeaaakeaaimaacqWFneVtaaa@383B@*P*-hard optimization problems is known as *stochastic local search *(SLS) [4]. Applied to protein folding problems, SLS methods attempt to find native states by iteratively performing small conformational modifications guided by the given energy function; randomized decisions are used to avoid getting trapped in local minima of the given energy landscape. Monte Carlo algorithms, which are widely used in protein structure prediction, are a prominent special case of SLS methods.

The performance of stochastic local search algorithms is critically dependent on the properties of the search landscape encountered, such as the number and distribution of local minima, the overall landscape ruggedness (measured, for example, using auto-correlation or fitness-distance analysis), and the basin structure. Therefore, search strategies that can extract important features of the landscape and adapt the search accordingly are among the most effective tools for solving optimization problems with complex search landscapes. As evident from an analysis of the literature describing search methods for *ab initio *protein folding presented in the related work section, such adaptive search strategies have not been widely studied for this problem.

In this work, we introduce a novel adaptive SLS method that is based on a system of bins for storing a diverse set of conformations (candidate solutions) in memory. The general idea behind our approach is to store promising conformations encountered during the search for later use in situations when search stagnation is detected. These conformations are pooled into a number of bins according to energy and diversity criteria. The storage and retrieval mechanisms used in this context adaptively control the behaviour of a subsidiary local search procedure, such as canonical Monte Carlo search, and are shown to greatly improve its performance.

The remainder of this paper is structured as follows: First, we provide background information and discuss related work on protein folding problems and algorithms. Next, we introduce the bin framework and describe a Monte Carlo algorithm that utilizes this bin framework adaptively. We then compare the performance of our algorithm with that of other prominent methods from the literature, followed by a discussion of how the behavior and performance of our algorithm is influenced by its parameters. Finally, we explain how the bin framework introduced in this work relates to other search methods known from the literature, summarize our findings, draw some general conclusions, and indicate directions for future work. In the "Methods" section, we describe the face-centered cubic (FCC) lattice model and *β*-sheet energy potential used in our computational analysis and provides details of the experimental protocols used in the empirical analysis of our algorithm.

### Related work

To address the *ab initio *protein folding problem, the following three issues need to be considered: (1) the model used for the representation of protein structure (which may have implications on prediction accuracy); (2) the energy potential function (which ideally should be able to discriminate between native and non-native conformations); and (3) the method used for searching through the space of possible conformations (which should be able to find optimal conformations as efficiently as possible). In this section, we discuss the choice of protein representation and energy function made in this work; we also provide a brief overview of the best-performing search methods for *ab initio *protein folding known from the literature.

To facilitate *ab initio *protein structure prediction by means of more efficient methods for searching in the space of protein conformations, various reduced models of protein structure have been introduced by biochemists and physicists. These reduced models fall into two major classes: lattice and off-lattice models. The primary reason for choosing off-lattice models over lattice models is to obtain better geometrical accuracy. Despite the biases introduced by the lattice models, namely a somewhat restricted ability to accurately represent secondary structure and backbone conformation, lattice models still retain essential properties of the system [5-7] and offer a number of computational advantages; these advantages include fast energy computation, easiness of testing self-avoidance and the ability to use pre-computed tables of moves, all of which help to compute search steps efficiently.

An important representative of lattice models is the Face-Centered Cubic (FCC) lattice, which underlies the crystalline structure of most metals. Even though in the context of *ab initio *protein structure prediction, the simpler square and cubic lattices are the most widely studied models in the literature, the FCC lattice captures real protein conformations with higher accuracy (coordinate *C*_*α *_root mean square deviation below 2 Å) [7] while still being representationally rather simple (it requires only 12 basis vectors). It has also been shown that local packing of amino acids in real proteins closely fits a distorted FCC lattice [8], and that the FCC model allows a reasonable description of secondary structure elements; furthermore, it can represent geometrically accurate hydrogen bonding [6]. As a result, the FCC lattice is considered the best overall choice among the simpler regular lattice models [6]. A detailed description of the FCC lattice is provided in the "Methods" Section.

As an energy function to be used in conjunction with the FCC lattice model we chose the *β*-sheet potential [9,10]. This choice was motivated by the fact that there are no universally used energy functions for *ab initio *protein structure prediction; at the same time, the *β*-sheet potential has been used relatively widely in the literature for the empirical evaluation of the best-performing protein structure prediction methods discussed later in this section. This enables us to compare our new approach against a relatively wide range of other conformation search methods known from the literature. Furthermore, this *β*-sheet potential exhibits characteristics of more complicated energy functions used for off-lattice models [9,11] (particularly, cooperative all-or-none folding transition, characteristic interplay between short- and long-interactions and secondary structure propensity). Finally, the problem of folding of *β*-sheet proteins is particularly important, since the accuracy of protein structure prediction methods for *β*-sheet proteins is the lowest among all structural classes of proteins [12]. The *β*-sheet potential used in this work is described in detail in the "Methods" Section.

There are a number of search methods applicable to the protein folding problem that can be used in conjunction with reduced complexity models and simplified potentials to perform a broad search through low-resolution structures. The most widely used methods include Metropolis Monte Carlo (MC) search [13-17], Genetic Algorithms [18-20] and Generalized Ensemble Methods [21-23], which include the currently best-performing algorithms for *ab initio *protein structure prediction. This last class of algorithms is based on the observation that canonical Monte Carlo methods sample conformations according to Boltzmann probabilities. For typical distributions of states (*i*.*e*., protein conformations) over energy levels this means that very high and, more importantly, very low energy conformations are rarely sampled. Generalized Ensemble Monte Carlo Methods attempt to overcome this problem by striving to perform a random walk in energy space by computing the density of states, by sampling expanded range of temperatures or by computing other physical quantities affecting transitions between the states during search.

Currently, the best-performing Generalized Ensemble Method for *ab initio *protein structure prediction is Replica Exchange Monte Carlo search (REMC) [23], also known as the multiple Markov Chain method or Parallel Tempering [22]. In REMC, a number of non-interacting copies (replicas) of the given protein sequence are folded independently and at different temperature settings of the underlying canonical Monte Carlo search. Every few steps, pairs of replicas are exchanged (*i*.*e*., the temperature settings of the MC search performed on them are swapped) with a probability that depends on the energies of the respective conformations (using Boltzmann weighting). While other Generalized Ensemble Methods, such as Multicanonical (MUCA) Monte Carlo (or Entropy Sampling Monte Carlo) search [9], maintain only one conformation at any given time, the number of replicas required in REMC increases as the square root of the number of degrees of freedom (which in its turn increases linearly with sequence length) [23].

Improvements of REMC introduced in the literature include hybrid approaches between REMC (for the weight factor determination) and MUCA, or Simulated Tempering production runs [22-24]. The Parallel-Hat Tempering (PHAT) Monte Carlo method utilizes an additional weight factor based on the histogram of energies sampled by each temperature replica [10] to achieve an exponential increase in the acceptance probabilities for high- and low-energy conformations, which increases the efficiency of the search process by allowing it to effectively overcome higher energy barriers and to explore a wider range of conformations for each replica.

The following algorithms have been implemented and empirically evaluated for the FCC lattice model with the *β*-sheet energy function considered in this work: canonical Metropolis Monte Carlo search [9], MUCA [9], REMC [9] and PHAT [10]. Of these, PHAT is the best-performing conformation search method for the *ab initio *prediction of protein structures on the FCC lattice using the *β*-sheet potential.

### Bin framework Monte Carlo search

The key idea behind our adaptive bin framework is to improve the effectiveness of a given search process, such as canonical Monte Carlo search, by making it adaptive and augmenting it with a mechanism for storing and retrieving promising conformations. This is achieved by using a series of bins each of which holds a set of conformations within a certain energy range and an adaptive strategy for restarting a given search process with a conformation retrieved from these bins when the search stagnates. By varying the search strategy according to *a priori *defined transition probabilities (which are dependent on the search progress), this approach leads to an algorithm that sacrifices an exact relationship with the canonical ensemble for search efficiency. This method effectively reduces the slow convergence, or quasi-ergodicity, in rugged energy landscapes; it is therefore very useful when the main interest is in finding global minima, rather than in obtaining other physical properties from canonical ensembles.

Conformations are added to the bins in a way that is aimed at maintaining a diverse set of low-energy conformations. To achieve this we define energy and diversity thresholds for each bin, which are dynamically modified during the search process (it may be noted that this adaptive strategy is closely related to the concept of reactive search [25]).

With each bin *i *the following properties are associated:

• The capacity of the bin, *cap*_*i*_, *i.e*., the maximal number of conformations that can be stored in the bin at any given time.

• The current number of conformations stored in the bin, *num*_*i*_. These conformations themselves are stored in a list that is sorted according to energy, to facilitate efficient retrieval of low-energy conformations.

• The width of the bin's maximal energy range, Δ*E*_*i*_.

• The bin's energy threshold, Ei+
 MathType@MTEF@5@5@+=feaafiart1ev1aaatCvAUfKttLearuWrP9MDH5MBPbIqV92AaeXatLxBI9gBaebbnrfifHhDYfgasaacH8akY=wiFfYdH8Gipec8Eeeu0xXdbba9frFj0=OqFfea0dXdd9vqai=hGuQ8kuc9pgc9s8qqaq=dirpe0xb9q8qiLsFr0=vr0=vr0dc8meaabaqaciaacaGaaeqabaqabeGadaaakeaacqWGfbqrdaqhaaWcbaGaemyAaKgabaGaey4kaScaaaaa@3029@. This is the highest energy that a conformation can reach and still be placed into bin *i *if the respective diversity threshold (described in the following) is satisfied.

• The diversity threshold, which determines how different a conformation has to be from other conformations already stored at the same energy level in order for the new conformation to be accepted into the bin. The pairwise diversity of conformations is measured using a distance measure that depends on the protein model under consideration. Here, we use the normalized average Hamming distance, *HD*, between the *β*-sheet energy sequence of a newly considered conformation *c *and all *β*-sheet energy sequences for the set *C' *of all conformations with the same energy that are already in the bin, see Methods Section for details.

Furthermore, the overall bin framework has the following parameters:

• The total number of bins, *numBins*.

• The energy range of interest, Δ*E*. Together with the current estimate of the ground state energy, E^
 MathType@MTEF@5@5@+=feaafiart1ev1aaatCvAUfKttLearuWrP9MDH5MBPbIqV92AaeXatLxBI9gBaebbnrfifHhDYfgasaacH8akY=wiFfYdH8Gipec8Eeeu0xXdbba9frFj0=OqFfea0dXdd9vqai=hGuQ8kuc9pgc9s8qqaq=dirpe0xb9q8qiLsFr0=vr0=vr0dc8meaabaqaciaacaGaaeqabaqabeGadaaakeaacuWGfbqrgaqcaaaa@2DCF@, which is modified throughout the search to always represent the lowest energy encountered so far, Δ*E *controls the energy interval into which conformations must fall in order to be accepted into any bin. This range represents the estimate of the maximal barrier height that needs to be surmounted to reach lower energy conformations.

• The temperature *T*_*bin*_, which controls the retrieval of conformations from bins.

**Figure 1:**
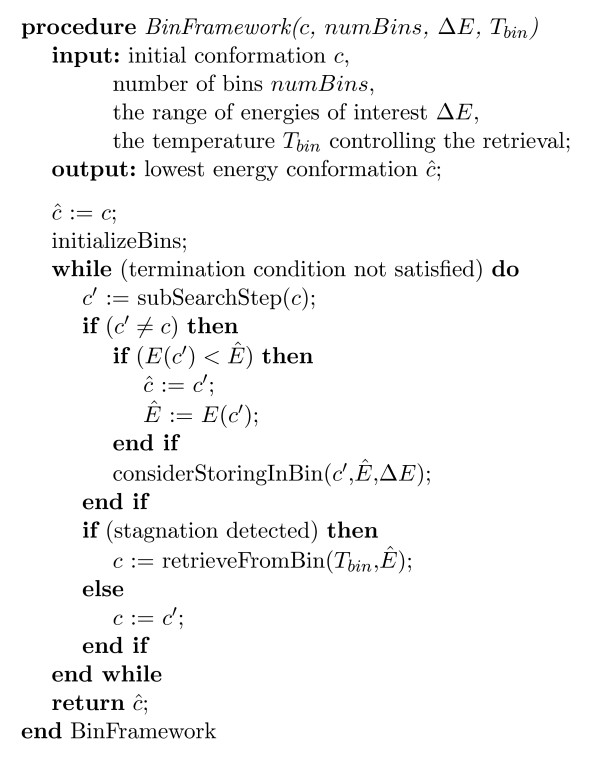
**High-level outline of the main body of the Bin Framework Search Method**. Outline of the main body of the Bin Framework Search Method.

The general bin framework search mechanism is outlined in Figure 1. Procedure *initalizeBins *is used to set all bin parameters to their initial values. Bins are numbered 1 ... *numBins*, and for every bin *i*, Δ*E*_*i *_and Ei+
 MathType@MTEF@5@5@+=feaafiart1ev1aaatCvAUfKttLearuWrP9MDH5MBPbIqV92AaeXatLxBI9gBaebbnrfifHhDYfgasaacH8akY=wiFfYdH8Gipec8Eeeu0xXdbba9frFj0=OqFfea0dXdd9vqai=hGuQ8kuc9pgc9s8qqaq=dirpe0xb9q8qiLsFr0=vr0=vr0dc8meaabaqaciaacaGaaeqabaqabeGadaaakeaacqWGfbqrdaqhaaWcbaGaemyAaKgabaGaey4kaScaaaaa@3029@ are always assigned such that Ei+<Ei−1+−ΔEi−1+1
 MathType@MTEF@5@5@+=feaafiart1ev1aaatCvAUfKttLearuWrP9MDH5MBPbIqV92AaeXatLxBI9gBaebbnrfifHhDYfgasaacH8akY=wiFfYdH8Gipec8Eeeu0xXdbba9frFj0=OqFfea0dXdd9vqai=hGuQ8kuc9pgc9s8qqaq=dirpe0xb9q8qiLsFr0=vr0=vr0dc8meaabaqaciaacaGaaeqabaqabeGadaaakeaacqWGfbqrdaqhaaWcbaGaemyAaKgabaGaey4kaScaaOGaeyipaWJaemyrau0aa0baaSqaaiabdMgaPjabgkHiTiabigdaXaqaaiabgUcaRaaakiabgkHiTiabfs5aejabdweafnaaBaaaleaacqWGPbqAcqGHsislcqaIXaqmaeqaaOGaey4kaSIaeGymaedaaa@3F41@, *i.e*., the energy intervals [Ei+,Ei+−ΔEi)
 MathType@MTEF@5@5@+=feaafiart1ev1aaatCvAUfKttLearuWrP9MDH5MBPbIqV92AaeXatLxBI9gBaebbnrfifHhDYfgasaacH8akY=wiFfYdH8Gipec8Eeeu0xXdbba9frFj0=OqFfea0dXdd9vqai=hGuQ8kuc9pgc9s8qqaq=dirpe0xb9q8qiLsFr0=vr0=vr0dc8meaabaqaciaacaGaaeqabaqabeGadaaakeaacqGGBbWwcqWGfbqrdaqhaaWcbaGaemyAaKgabaGaey4kaScaaOGaeiilaWIaemyrau0aa0baaSqaaiabdMgaPbqaaiabgUcaRaaakiabgkHiTiabfs5aejabdweafnaaBaaaleaacqWGPbqAaeqaaOGaeiykaKcaaa@3BA9@ for different bins never overlap.

Furthermore, the energy bounds are always assigned such that Ei+>E2+>⋯>EnumBins+
 MathType@MTEF@5@5@+=feaafiart1ev1aaatCvAUfKttLearuWrP9MDH5MBPbIqV92AaeXatLxBI9gBaebbnrfifHhDYfgasaacH8akY=wiFfYdH8Gipec8Eeeu0xXdbba9frFj0=OqFfea0dXdd9vqai=hGuQ8kuc9pgc9s8qqaq=dirpe0xb9q8qiLsFr0=vr0=vr0dc8meaabaqaciaacaGaaeqabaqabeGadaaakeaacqWGfbqrdaqhaaWcbaGaemyAaKgabaGaey4kaScaaOGaeyOpa4Jaemyrau0aa0baaSqaaiabikdaYaqaaiabgUcaRaaakiabg6da+iabl+Uimjabg6da+iabdweafnaaDaaaleaacqWGUbGBcqWG1bqDcqWGTbqBcqWGcbGqcqWGPbqAcqWGUbGBcqWGZbWCaeaacqGHRaWkaaaaaa@43F0@, *i.e*., bin 1 always has the highest energy interval, while bin *numBins *stores the lowest energy conformations. Procedure *subSearchStep *performs one step of a subsidiary search procedure (such as canonical MC search) on a given conformation *c *and returns the resulting conformation *c'*. This step involves a single proposal of the move from the given move set and its subsequent acceptance or rejection. The two procedures *considerStoringInBin *and *retrieveFromBin *control the storage of conformations in the bin system and the retrieval of previously stored conformations. Note that conformations will only be stored in a bin if they satisfy the corresponding energy and diversity thresholds. Storing a conformation may lead to adjustments of the energy thresholds for the corresponding bin or addition of a new bin (this will be described later in detail for the BINMC algorithm). Finally, a stagnation criterion is used to decide when to retrieve a conformation from the bin system in order to overcome search stagnation, and a termination condition is used to determine when the search process should terminate.

In the following, we will describe the specific instantiation of this framework on which the remainder of our study is focused: the BINMC algorithm.

In BINMC, for simplicity all bin capacities *cap*_*i *_are set to the same value, and this value is kept constant during the search. The same holds for the energy ranges Δ*E*_*i*_. Finally, for simplicity we also keep the number of bins, *numBins*, constant during the run of the algorithm. This number is determined by the interval of energies of interest and the energy window width used:

*numBins *= ⌈Δ*E*/Δ*E*_*i*_⌉.

At the beginning of the search process, the energy threshold for each bin *i *is set to Ei+
 MathType@MTEF@5@5@+=feaafiart1ev1aaatCvAUfKttLearuWrP9MDH5MBPbIqV92AaeXatLxBI9gBaebbnrfifHhDYfgasaacH8akY=wiFfYdH8Gipec8Eeeu0xXdbba9frFj0=OqFfea0dXdd9vqai=hGuQ8kuc9pgc9s8qqaq=dirpe0xb9q8qiLsFr0=vr0=vr0dc8meaabaqaciaacaGaaeqabaqabeGadaaakeaacqWGfbqrdaqhaaWcbaGaemyAaKgabaGaey4kaScaaaaa@3029@ := -(*i *- 1)·Δ*E*_*i*_. Initially, the energy intervals of all bins form a partition of the interval [0, *numBins*·Δ*E*_*i*_), note that this interval is larger than or equal to the desired interval [0, Δ*E*); it is larger if Δ*E *does not divide evenly by Δ*E*_*i*_.

It should be noted that 0 is the highest energy possible under the model chosen in this work and all the energies are integer values; in the general case energy thresholds can be adjusted initially to store the highest energy conformations under the protein model considered. The bin energy bounds are adjusted during the search, as will be explained later.

**Figure 2:**
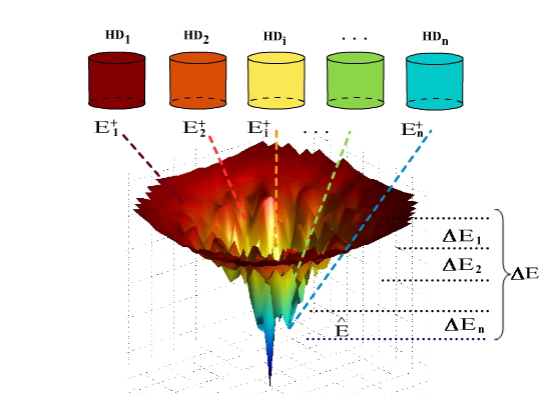
**Relationship between the bin framework and the energy landscape**. An illustration of how conformations at a given state of the bin framework relate to the energy landscape of a given protein. E^
 MathType@MTEF@5@5@+=feaafiart1ev1aaatCvAUfKttLearuWrP9MDH5MBPbIqV92AaeXatLxBI9gBaebbnrfifHhDYfgasaacH8akY=wiFfYdH8Gipec8Eeeu0xXdbba9frFj0=OqFfea0dXdd9vqai=hGuQ8kuc9pgc9s8qqaq=dirpe0xb9q8qiLsFr0=vr0=vr0dc8meaabaqaciaacaGaaeqabaqabeGadaaakeaacuWGfbqrgaqcaaaa@2DCF@ is the best solution quality found so far and serves as an estimate of the ground state energy, Δ*E *is the energy range of interest, and conformations within this range are binned. Each bin *i *has energy threshold Ei+
 MathType@MTEF@5@5@+=feaafiart1ev1aaatCvAUfKttLearuWrP9MDH5MBPbIqV92AaeXatLxBI9gBaebbnrfifHhDYfgasaacH8akY=wiFfYdH8Gipec8Eeeu0xXdbba9frFj0=OqFfea0dXdd9vqai=hGuQ8kuc9pgc9s8qqaq=dirpe0xb9q8qiLsFr0=vr0=vr0dc8meaabaqaciaacaGaaeqabaqabeGadaaakeaacqWGfbqrdaqhaaWcbaGaemyAaKgabaGaey4kaScaaaaa@3029@, diversity threshold *HD*_*i*_, and energy window Δ*E*_*i*_.

The diversity thresholds for the bins, *HD*_*i*_, are determined based on the following formula:

*HD*_*i *_= *HD*_*min *_+ (*numBins *- *i*)·(*HD*_*max *_- *HD*_*min*_)/(*numBins *- 1),

where *HD*_*min *_and *HD*_*max *_are parameters of the algorithm that determine the diversity threshold of the lowest and highest energy bins, respectively. This choice is based on the experimental observation that fewer protein conformations exist for lower energies, and therefore, the set of of conformations to be found at low energy levels can be expected to be less diverse. (This is also consistent with the prevalent view that the energy landscapes encountered in *ab initio *protein structure prediction problems are funneled [26].) Figure 2 depicts some of the properties of bins and conformations in a bin and the overall relationship of conformations stored in the framework to the energy landscape.

BINMC uses a standard canonical Monte Carlo search procedure running at a constant inverse temperature *β*_*MC *_:= 1/(*k*_*B*_·*T*_*MC*_) (where *k*_*B *_denotes the Boltzmann constant), that controls the probability with which worsening search steps are accepted. Our canonical Monte Carlo procedure for the FCC lattice is based on the same search neighbourhood (move set) as used by Gront *et al*. [9] and by Zhang and Skolnick [10]. In each search step, either a double-bond move or a chain-end move is attempted. A double-bond move involves the modification of two successive bond angles, whose position in the chain is chosen uniformly at random. Similarly, in a chain-end move, the location of the first or the last residue is changed. For efficiency and speed, double-bond moves are pre-computed in a table, as done by Gront *et al*. [9] and by Zhang & Skolnick [10]. The search proceeds in phases, each of which starts with two attempts at chain-end moves (one on each end, in random order) followed by *N*/2 successive attempts at double-bond moves (each chosen uniformly at random, without repetition but allowing overlap with previously chosen double-bond moves) any number of which may be accepted. Procedure *subSearchStep *in Figure 1 performs a single step of this simple subsidiary search process by attempting one move, starting from conformation *c *and resulting in conformation *c' *(which, if the proposed move has not been accepted, is equal to *c*); the attempted moves are chosen such that the previously described phasing of chain-end and double-bond moves is ensured.

After a new conformation has been accepted by the subsidiary MC procedure, it is considered for placement into a bin. If the new conformation *c' *has lower energy then any other conformation encountered so far, *i.e*., if *E*(*c'*) <E^
 MathType@MTEF@5@5@+=feaafiart1ev1aaatCvAUfKttLearuWrP9MDH5MBPbIqV92AaeXatLxBI9gBaebbnrfifHhDYfgasaacH8akY=wiFfYdH8Gipec8Eeeu0xXdbba9frFj0=OqFfea0dXdd9vqai=hGuQ8kuc9pgc9s8qqaq=dirpe0xb9q8qiLsFr0=vr0=vr0dc8meaabaqaciaacaGaaeqabaqabeGadaaakeaacuWGfbqrgaqcaaaa@2DCF@, it is always accepted into the bin framework and the current estimate of the ground state, E^
 MathType@MTEF@5@5@+=feaafiart1ev1aaatCvAUfKttLearuWrP9MDH5MBPbIqV92AaeXatLxBI9gBaebbnrfifHhDYfgasaacH8akY=wiFfYdH8Gipec8Eeeu0xXdbba9frFj0=OqFfea0dXdd9vqai=hGuQ8kuc9pgc9s8qqaq=dirpe0xb9q8qiLsFr0=vr0=vr0dc8meaabaqaciaacaGaaeqabaqabeGadaaakeaacuWGfbqrgaqcaaaa@2DCF@, is updated. If *E*(*c'*) falls outside the energy range currently represented by the bin framework, before accepting the new conformation, a new bin (or a number of bins if needed) is created and the first bin storing conformations with high energies (or a number of bins starting with the first one) is deleted as follows: (here we only describe addition of a single bin, addition of multiple bins is handled analogously): We add a new bin *numBins *+ 1 and delete all conformations from bin 1 along with bin 1 itself. We also shift bin numbers by -1, such that bin 2 becomes bin 1 and bin *numBins *+ 1 becomes bin *numBins*. The energy threshold EnumBins+
 MathType@MTEF@5@5@+=feaafiart1ev1aaatCvAUfKttLearuWrP9MDH5MBPbIqV92AaeXatLxBI9gBaebbnrfifHhDYfgasaacH8akY=wiFfYdH8Gipec8Eeeu0xXdbba9frFj0=OqFfea0dXdd9vqai=hGuQ8kuc9pgc9s8qqaq=dirpe0xb9q8qiLsFr0=vr0=vr0dc8meaabaqaciaacaGaaeqabaqabeGadaaakeaacqWGfbqrdaqhaaWcbaGaemOBa4MaemyDauNaemyBa0MaemOqaiKaemyAaKMaemOBa4Maem4CamhabaGaey4kaScaaaaa@3845@ for the newly added bin is set to EnumBins−1+−ΔEnumBins−1+1
 MathType@MTEF@5@5@+=feaafiart1ev1aaatCvAUfKttLearuWrP9MDH5MBPbIqV92AaeXatLxBI9gBaebbnrfifHhDYfgasaacH8akY=wiFfYdH8Gipec8Eeeu0xXdbba9frFj0=OqFfea0dXdd9vqai=hGuQ8kuc9pgc9s8qqaq=dirpe0xb9q8qiLsFr0=vr0=vr0dc8meaabaqaciaacaGaaeqabaqabeGadaaakeaacqWGfbqrdaqhaaWcbaGaemOBa4MaemyDauNaemyBa0MaemOqaiKaemyAaKMaemOBa4Maem4CamNaeyOeI0IaeGymaedabaGaey4kaScaaOGaeyOeI0IaeuiLdqKaemyrau0aaSbaaSqaaiabd6gaUjabdwha1jabd2gaTjabdkeacjabdMgaPjabd6gaUjabdohaZjabgkHiTiabigdaXaqabaGccqGHRaWkcqaIXaqmaaa@4AEE@. The diversity thresholds are not shifted with the bins, such that *HD*_*max *_and *HD*_*min *_remain associated with the first and the last bin, respectively.

If conformation *E*(*c'*) falls within the energy interval of a bin *i *that is not yet filled to capacity (*i.e*., *num*_*i *_*< cap*_*i*_), and *c' *satisfies the diversity criterion for that bin – *i.e*., the Hamming distance between the conformation *c' *and other conformations *c" *with the same energy *E *should be larger or equal to *HD*_*i *_(see Methods Section for details) – *c' *is added to that bin, and *num*_*i *_is incremented by one. (Note that there is at most one such bin *i*, since bin energy intervals never overlap.) Finally, if *E*(*c'*) falls within the energy interval of a bin *i *that is already filled to capacity (*i.e*., *num*_*i *_= *cap*_*i*_) and it satisfies the diversity criterion for that bin, *c' *is added to the content of bin *i *and the highest energy conformation is removed. At the same time, E^
 MathType@MTEF@5@5@+=feaafiart1ev1aaatCvAUfKttLearuWrP9MDH5MBPbIqV92AaeXatLxBI9gBaebbnrfifHhDYfgasaacH8akY=wiFfYdH8Gipec8Eeeu0xXdbba9frFj0=OqFfea0dXdd9vqai=hGuQ8kuc9pgc9s8qqaq=dirpe0xb9q8qiLsFr0=vr0=vr0dc8meaabaqaciaacaGaaeqabaqabeGadaaakeaacuWGfbqrgaqcaaaa@2DCF@ is set to the energy of the conformation that is currently the highest in the bin; as a consequence, conformations with energy above the updated E^
 MathType@MTEF@5@5@+=feaafiart1ev1aaatCvAUfKttLearuWrP9MDH5MBPbIqV92AaeXatLxBI9gBaebbnrfifHhDYfgasaacH8akY=wiFfYdH8Gipec8Eeeu0xXdbba9frFj0=OqFfea0dXdd9vqai=hGuQ8kuc9pgc9s8qqaq=dirpe0xb9q8qiLsFr0=vr0=vr0dc8meaabaqaciaacaGaaeqabaqabeGadaaakeaacuWGfbqrgaqcaaaa@2DCF@ will not be accepted into bin *i *in the future, and therefore, the energy ranges of bins *i *- 1 and *i *are now may no longer be adjacent.

The stagnation detection mechanism used in BINMC is quite simple: the search is considered to be stagnated when no improvement on the lowest energy has been achieved for *noImprRetrieve *search steps, where *noImprRetrieve *is a parameter of the algorithm.

To retrieve a conformation from the bin system, BINMC uses a two-phase procedure that first selects a bin and then chooses one of the conformations stored in that bin. In the first phase, the probability of selecting a bin *i *depends on the difference between its upper energy threshold E^
 MathType@MTEF@5@5@+=feaafiart1ev1aaatCvAUfKttLearuWrP9MDH5MBPbIqV92AaeXatLxBI9gBaebbnrfifHhDYfgasaacH8akY=wiFfYdH8Gipec8Eeeu0xXdbba9frFj0=OqFfea0dXdd9vqai=hGuQ8kuc9pgc9s8qqaq=dirpe0xb9q8qiLsFr0=vr0=vr0dc8meaabaqaciaacaGaaeqabaqabeGadaaakeaacuWGfbqrgaqcaaaa@2DCF@ and the best energy reached so far, and is proportional to:

e−βbin(Ei+−E^)
 MathType@MTEF@5@5@+=feaafiart1ev1aaatCvAUfKttLearuWrP9MDH5MBPbIqV92AaeXatLxBI9gBaebbnrfifHhDYfgasaacH8akY=wiFfYdH8Gipec8Eeeu0xXdbba9frFj0=OqFfea0dXdd9vqai=hGuQ8kuc9pgc9s8qqaq=dirpe0xb9q8qiLsFr0=vr0=vr0dc8meaabaqaciaacaGaaeqabaqabeGadaaakeaacqWGLbqzdaahaaWcbeqaaiabgkHiTGGaciab=j7aInaaBaaameaacqWGIbGycqWGPbqAcqWGUbGBaeqaaSGaeiikaGIaemyrau0aa0baaWqaaiabdMgaPbqaaiabgUcaRaaaliabgkHiTiqbdweafzaajaGaeiykaKcaaaaa@3C51@

where *β*_*bin *_= 1/(*k*_*B*_·*T*_*bin*_), *k*_*B *_denotes the Boltzmann constant, and *T*_*bin *_is BINMC's temperature parameter. In the second phase, the probability of choosing a conformation *c *from that bin is analogously proportional to:

e−βbin⋅(E(c)−E^)
 MathType@MTEF@5@5@+=feaafiart1ev1aaatCvAUfKttLearuWrP9MDH5MBPbIqV92AaeXatLxBI9gBaebbnrfifHhDYfgasaacH8akY=wiFfYdH8Gipec8Eeeu0xXdbba9frFj0=OqFfea0dXdd9vqai=hGuQ8kuc9pgc9s8qqaq=dirpe0xb9q8qiLsFr0=vr0=vr0dc8meaabaqaciaacaGaaeqabaqabeGadaaakeaacqWGLbqzdaahaaWcbeqaaiabgkHiTGGaciab=j7aInaaBaaameaacqWGIbGycqWGPbqAcqWGUbGBaeqaaSGaeyyXICTaeiikaGIaemyrauKaeiikaGIaem4yamMaeiykaKIaeyOeI0IafmyrauKbaKaacqGGPaqkaaaaaa@3F26@

In general, conformations could be chosen with or without replacement; here we limited ourselves to choosing conformation with replacement, since the same conformation can yield a different fold each time it is picked.

As in the stochastic tunneling approach [27], to lessen exponential decay of the probability function we used Boltzmann-based modified weights proportional to e−β(E−E^)
 MathType@MTEF@5@5@+=feaafiart1ev1aaatCvAUfKttLearuWrP9MDH5MBPbIqV92AaeXatLxBI9gBaebbnrfifHhDYfgasaacH8akY=wiFfYdH8Gipec8Eeeu0xXdbba9frFj0=OqFfea0dXdd9vqai=hGuQ8kuc9pgc9s8qqaq=dirpe0xb9q8qiLsFr0=vr0=vr0dc8meaabaqaciaacaGaaeqabaqabeGadaaakeaacqWGLbqzdaahaaWcbeqaaiabgkHiTGGaciab=j7aIjabcIcaOiabdweafjabgkHiTiqbdweafzaajaGaeiykaKcaaaaa@3596@. This weighting preserves the location of all minima, but maps the entire energy space from E^
 MathType@MTEF@5@5@+=feaafiart1ev1aaatCvAUfKttLearuWrP9MDH5MBPbIqV92AaeXatLxBI9gBaebbnrfifHhDYfgasaacH8akY=wiFfYdH8Gipec8Eeeu0xXdbba9frFj0=OqFfea0dXdd9vqai=hGuQ8kuc9pgc9s8qqaq=dirpe0xb9q8qiLsFr0=vr0=vr0dc8meaabaqaciaacaGaaeqabaqabeGadaaakeaacuWGfbqrgaqcaaaa@2DCF@ to the maximum energy 0 onto the interval [0, 1]. The dynamic process following the Boltzmann distribution can therefore pass through energy barriers of an arbitrary height.

Overall, this retrieval procedure ensures that lower energy conformations are selected with higher probability, which is in accordance with the belief that the energy landscapes of real proteins are funneled. The search is terminated when the target energy level has been reached or when a user-specified number of steps has been executed.

## Results

In this section, we present empirical performance results for BINMC as compared to the best-performing algorithms known from the literature. MC, REMC and PHAT have been tested by their original authors on the homopolymers of length 32 and 64, but only results for the homopolymer of length 64 have been published [9,10]. (We contacted D. Gront [9] and Y. Zhang [10], who commented that all of their algorithms were able to reach quite easily what they believed to be the global minimum for the polymer of length 32. However, they could not provide any information regarding the energy values or conformations reached in these experiments.) For the protein of length 64, Gront *et al*. believed the lowest energy they had reached, -374, to correspond to a ground state conformation; however, Zhang and Skolnick later reported a conformation with energy -387 for this system [10].

Analogous with the results of Gront *et al*. for MC and REMC [9] and of Zhang and Skolnick for PHAT [10], in Table 1 we provide results averaged over 10 independent runs of each algorithm. It should be noted that several details were not evident from the published descriptions of these algorithms. From personal communication with D. Gront, we learned that their MC procedure was run with temperatures from 2.75 to 1.25 [*ε*_0_/*k*_*B*_]. Since we could not determine the exact annealing schedule used in their study, we chose a constant temperature of 1.25 for our MC procedure. Therefore, the results for the MCSA algorithm of Gront *et al*. may not be exactly comparable with that of our implementation of pure MC. Since the number of iterations performed within a given amount of time varies significantly based on implementation details, we used the average CPU times reported by Gront *et al*. [9] and by Zhang and Skolnick [10] as the cut-off time for our algorithms.

**Table 1:** Performance differences among algorithms for the homopolymer of length 64.

Method	Temperature set	*Time*_*cut*-*off*_	*E*_*avg *_± *sd*	*E*_*min*_	*P – value*
MCSA [9]	annealed from 2.75 to 1.25	24 min (approx)	-349.3 (± 2.1)	-362	
REMC [9]	linear 1.25 to 2.75	28 min (approx)	-368.2 (± 0.8)	-373	
PHAT [10]	linear 1.25 to 2.75	1 hr 25 min (approx)	**-380.4 **(± 1.9)	-387	

our MC	1.25	24 min	-367.2 (± 1.7)	-370	

our MC	1.25	28 min	-367.4 (± 2.7)	-371	0.1367
our REMC	linear 1.25 to 2.75	28 min	-368.5 (± 2.1)	-373	0.3425
our PHAT	linear 1.3 to 2.75	28 min	-367.5 (± 3.3)	-372	0.1599
our BINMC	*T*_*MC *_= 1.25, *T*_*bin *_= 6.521	28 min	**-370.3 **(± 4.3)	-379	

our MC	1.25	1 hr 25 min	-368.2 (± 4.6)	-374	0.0006*
our REMC	linear 1.25 to 2.75	1 hr 25 min	-369.4 (± 3.0)	-376	0.0008*
our PHAT	linear 1.3 to 2.75	1 hr 25 min	-369.5 (± 3.2)	-376	0.0023*
our BINMC	*T*_*MC *_= 1.25, *T*_*bin *_= 6.521	1 hr 25 min	**-375.7 **(± 3.8)	-383	

Comparison of the energy levels reached for the homopolymer of length *N *= 64 by Monte Carlo Simulated Annealing (MCSA) [9], the Replica Exchange Monte Carlo (REMC) algorithm with a linear set of temperatures [9] and the Parallel-hat Tempering algorithm (PHAT) [10] with our implementation of Monte Carlo (MC), REMC and PHAT, as well as with our new Bin Framework Monte Carlo (BINMC) algorithm. The run-time reported for MCSA and REMC [9] has been conservatively adjusted to our 2.4 *GHz *reference machine (this was done by dividing the published run-times, which have been obtained on a 500 *MHz *CPU, by a factor of 4.8). The same was done for the run-times for PHAT (which were originally obtained on a 750 *MHz *CPU and therefore conservatively divided by 3.2).

As seen from Table 1, our implementations of MC and REMC show comparable performance to that of MCSA and REMC by Gront *et al*. [9]. (As discussed in the table caption, differences in execution environments were taken into account.) However, our implementation of PHAT did not reach the performance reported in the literature [10]. Therefore, we contacted Y. Zhang to check unpublished details of their algorithm, but unfortunately, those details along with the precise information on the best conformation reported in their paper (with energy -387) were no longer available due to data loss. Our novel BINMC algorithm performs better than MC and REMC, and than our implementation of PHAT. We used the following set of parameters for the bin framework for the homopolymer of length 64: Δ*E *= 30 [*ε*_0_], Δ*E*_*i *_= 5 [*ε*_0_], *T*_*MC *_= 1.25 [*ε*_0_/*k*_*B*_], *T*_*bin *_= 6.521 [*ε*_0_/*k*_*B*_], *binCapacity *= 100, *HD*_*MAX *_= 0.8, *HD*_*MIN *_= 0.01, *noImprRetrieve *= 2 000 000 steps. These settings were determined in a series of experiments in which we studied the influence of different parameter settings; these will be further discussed in Discussion Section.

**Figure 3:**
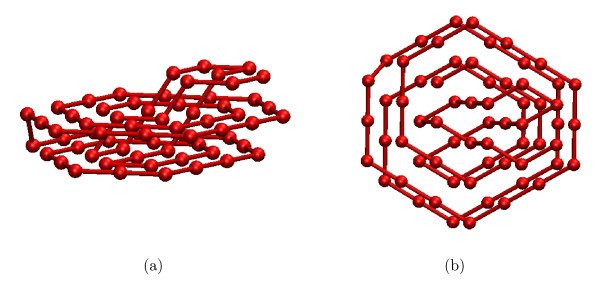
**The lowest energy conformation found by BINMC for the homopolymer of length 64**. Part (a) shows the lowest energy conformation of homopolymer of length 64 found by BINMC (total energy -391, short-range energy -212, long-range energy -179). A detailed description of this conformation is given in the supplementary material, see Additional file 1. Part (b) shows same conformation as seen from above.

The lowest energy level for the homopolymer of length 64 reached by our BINMC algorithm is -391; this is lower than the energy of any conformation previously reported in the literature. Conformations with energy -391 were found in 2 out of 10 runs, each with a 100 CPU hour cut-off on our reference machine, after 47 and 55 CPU hours, respectively. One of the two resulting conformations is shown in Figure 3, the other is its exact mirror image. Conformations with energies of -389 and -388 (some of which are shown in the supplementary material, see Additional file 1), were found multiple times by BINMC within a CPU time cut-off of 10 hours on our reference machines.

To extend our comparison of the re-implemented methods from the literature (MC, REMC and PHAT) with BINMC, we tested these methods on instances of length 12, 24 and 32. Again, we performed 10 independent runs of each algorithm on every problem instance, measuring the mean as well as the standard deviation of the energy levels reached after a run-time of 1 CPU hour. For BINMC when applied to the homopolymers of length 12 and 24, we used the following parameter settings: Δ*E *= 20 [*ε*_0_], Δ*E*_*i *_= 5 [*ε*_0_], *T*_*MC *_= 1.25 [*ε*_0_/*k*_*B*_], *T*_*bin *_= 4.344 [*ε*_0_/*k*_*B*_], *binCapacity *= 100, *HD*_*MAX *_= 0.6, *HD*_*MIN *_= 0.01 and *noImprRetrieve *= 100 000 steps, whereas on the homopolymer of length 32, we set the parameters to: Δ*E *= 20 [*ε*_0_], Δ*E*_*i *_= 5 [*ε*_0_], *T*_*MC *_= 1.25 [*ε*_0_/*k*_*B*_], *T*_*bin *_= 4.344 [*ε*_0_/*k*_*B*_], *binCapacity *= 100, *HD*_*MAX *_= 0.6, *HD*_*MIN *_= 0.01 and *noImprRetrieve *= 1 000 000 steps. (These parameter settings are discussed in Discussion Section and in the supplementary material, see Additional file 1.)

**Figure 4:**
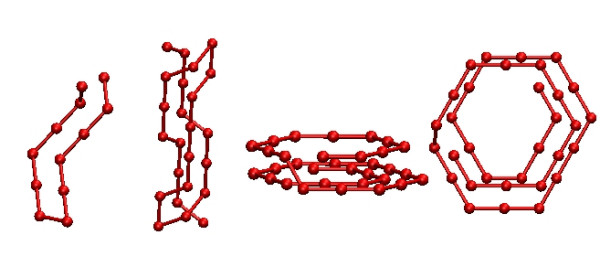
**Examples of the lowest energy conformations for the homopolymers of length 12, 24, and 32**. The lowest energy conformations of the FCC homopolymers of length 12, 24, and 32 amino acids (the last of these is shown from the side and above) found by the algorithms we tested; the corresponding energies are: -39 for *N *= 12 (short-range energy = -28, long-range energy = -11), -109 for *N *= 24 (short-range energy = -68, long-range energy = -41) and -161 for *N *= 32 (short-range energy = -112, long-range energy = -49). These conformations are specified in detail in the supplementary material, see Additional file 1.

As can be seen from our results presented in Table 2, all methods find what appears to be the lowest energy (-39) for the homopolymer of length 12 in less than 1 CPU second on our reference machine. For the homopolymer of length 24, we are starting to see differences among the algorithms: BINMC slightly outperforms all other algorithms in terms of CPU time required for reaching an energy of -109, which we believe to be the global minimum for this problem instance. MC is the next best method in terms of performance, followed by PHAT and REMC. The performance results for REMC and PHAT are worse than for MC because this homopolymer is too short for the additional time invested in exchanges between replicas to be amortized. For the homopolymer of length 32, BINMC outperforms the other algorithms by obtaining lower average energy (and also finding lower energy states, for example with energy -161, more often), followed by PHAT, REMC and finally MC. We show minimum energy conformations for the polymers of length 12, 24, and 32 in Figure 4. These solutions appear to be unique in terms of short-range and long-range energy values, since all of the conformations found by any of the algorithms we ran show the same short- *vs *long-range energy interplay.

**Table 2:** Performance differences among algorithms for the homopolymers of length 12, 24, 32.

Method	*Length*	*E*_*avg*_*± sd*	*E*_*min*_	*CPU Time*_*avg*_	*Time*_*med*_	*Time*_*q*75_	*Time*_*q*25_	*p – value*
MC	12	-39 (± 0)	-39	< 1 sec	< 1 sec	< 1 sec	< 1 sec	
REMC	12	-39 (± 0)	-39	< 1 sec	< 1 sec	< 1 sec	< 1 sec	
PHAT	12	-39 (± 0)	-39	< 1 sec	< 1 sec	< 1 sec	< 1 sec	
BINMC	12	-39 (± 0)	-39	< 1 sec	< 1 sec	< 1 sec	< 1 sec	

MC	24	-109 (± 0)	-109	5.0 min (± 4.1 min)	5.5 min	7.2 min	1.5 min	0.1230
REMC	24	-109 (± 0)	-109	18.3 min (± 18.0 min)	16.3 min	19.4 min	4.3 min	0.0015*
PHAT	24	-109 (± 0)	-109	8.7 min (± 8.2 min)	6.6 min	11.7 min	2.9 min	0.0039*
BINMC	24	-109 (± 0)	-109	**1.7 **min (± 1.2 min)	1.8 min	2.7 min	0.5 min	

Method	*Length*	*E*_*avg*_*± sd*	*Lowest E*	*CPU Time*_*avg*_	*E*_*med*_	*E *_*q*75_	*E *_*q*25_	*p – value*

MC	32	-158.1 (± 0.9)	-161	4.3 min (± 8.4 min)	-158	-158	-158	0.0155*
REMC	32	-158.2 (± 0.7)	-161	4.5 min (± 8.6 min)	-158	-158	-158	0.0185*
PHAT	32	-158.3 (± 0.9)	-161	5.8 min (± 8.0 min)	-158	-158	-158	0.0214*
BINMC	32	**-158.9 **(± 0.6)	-161	23.0 min (± 20.1 min)	-159	-159	-158	

Comparison of the average energy level obtained and the average time required for the homopolymers of lengths *N *= 12, 24, 32 for the re-implemented MC, REMC, PHAT, and BINMC. The time cut-off used was 1 CPU hour on our 2.4 *GHz *reference machine, and all statistics were calculated from 10 independent runs. The temperature sets used for our implementations of MC, REMC and are the same as in Table 1. The *p*-values reported in the last column were determined using the Mann-Whitney U test to test the null hypothesis that the mean run-time (for *N *= 24) and the mean energies reached by the respective algorithm *vs *BINMC (within the same CPU cut-off time for *N *= 32), respectively, are identical [4]; *p*-values marked with an asterisk (*) correspond to cases in which the null hypothesis is rejected at a standard significance level of 0.05, and therefore indicate statistically significant performance differences.

Additionally, to see to which extent the results reported in Tables 1 and 2 could be further improved, we carried out 10 long independent runs for the homopolymers of length 64 and 32, using a cut-off time of 10 CPU hours on our reference machine. The results of this experiment are shown in Table 3; clearly, BINMC outperforms our implementation of the state-of-the-art REMC and PHAT algorithms as well as the canonical MC algorithm in terms of the solution quality reached in these long runs, with REMC ranking second, followed by PHAT and MC.

**Table 3:** Performance differences among algorithms for the homopolymers of length 64 and 32 in long runs.

Method	Temperature set	*Length*	*E*_*avg*_*± sd*	*E*_*med*_	*E *_*q*75_	*E *_*q*25_	*E*_*min*_	*p – value*
our MC	1.25	32	-158.7 (± 1.9)	-159	-159	-158	-161	0.0271*
our REMC	linear 1.25 to 2.75	32	-159.6 (± 1.3)	-160	-161	-158	-161	0.5471
our PHAT	linear 1.3 to 2.75	32	-158.9 (± 1.4)	-159	-159	-158	-161	0.0638
our BINMC	*T*_*MC *_= 1.25, *T*_*bin *_= 6.521	32	**-160.1 **(± 0.9)	-161	-161	-159	-161	

our MC	1.25	64	-372.2 (± 2.3)	-372	-373	-371	-377	0.0005*
our REMC	linear 1.25 to 2.75	64	-376.1 (± 3.5)	-376	-378	-373	-382	0.0521
our PHAT	linear 1.3 to 2.75	64	-374.1 (± 3.8)	-374	-377	-371	-383	0.0120*
our BINMC	*T*_*MC *_= 1.25, *T*_*bin *_= 6.521	64	**-379.5 **(± 3.3)	-381	-382	-376	-389	

Comparison of the energy levels reached for the homopolymers of length 64 and 32 by our implementations of MC, REMC (with the linear set of temperatures), PHAT, and the new BINMC algorithm in 10 independent runs of 10 CPU hours each on our 2.4 GHz reference machine. The *p*-values reported in the last column were determined using the Mann-Whitney U test to test the null hypothesis that the mean energies reached by the respective algorithm and BINMC (within the same CPU cut-off time) are identical [4]; *p*-values marked with an asterisk (*) correspond to cases in which the null hypothesis is rejected at a standard significance level of 0.05, and therefore indicate statistically significant performance differences.

**Figure 5:**
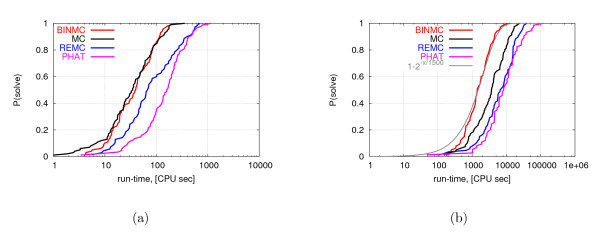
**Distribution of run-times for all algorithms required to reach sub-optimal conformations for homopolymers of length 32 and 64**. Distribution of run-times required by MC, REMC, PHAT and BINMC to reach sub-optimal conformations with energy -158 for the homopolymer of length 32 (part a) -370 for the homopolymer of length 64 (part b), based on 100 independent runs on our reference machine, each of which reached the target energy value. We fitted the run-time distribution (RTD) of BINMC for the homopolymer of length 64 with exponential distribution, to illustrate that the respective RTD is approximately exponential. (The same holds for all other RTDs shown in these plots.)

Next, we conducted a more thorough performance comparison of the algorithms based on run-time distributions (RTDs) measured on the homopolymers of length 32 and 64. The goal of this analysis was to analyze the variability between independent runs on the same problem instance, which is known to reflect the parallelization efficiency of an algorithm and can also reveal detrimental stagnation behavior [4]. In order to be able to perform 100 independent runs of each algorithm on both sequences within a reasonable overall computation time using our reference machine, we used sub-optimal energy levels of -158 and -370 for *N *= 32 and *N *= 64, respectively. The resulting empirical RTDs are shown in Figure 5 in the form of the respective cumulative distribution functions. For the homopolymer of length 32, BINMC and MC outperform REMC and PHAT in terms of the time required to reach the sub-optimal solution quality of -158. For the homopolymer of length 64 BINMC performs best in terms of the time required to reach the sub-optimal solution quality of -370, followed by MC, REMC and PHAT.

As can be seen from the graphs in Figure 5, the RTDs of all four algorithms closely resemble exponential distributions. (Note that the cumulative distribution functions of all exponential distributions have exactly the same shape when shown in a semi-log plot.) This indicates that for reaching the energy levels considered here, none of the algorithms stagnates and all of them can be parallelized efficiently by concurrently executing independent runs [4].

It may come as a surprise that MC performs better than REMC and PHAT. However, it is important to note that the RTDs reported in Figure 5 are for sub-optimal qualities only. Since MC at a low temperature is "greedier" and does not run multiple chains at different temperatures nor attempts exchanges between them, it gets to sub-optimal energies faster. After reaching them, however, it stagnates; this is reflected in the observation that solution quality does not improve when running MC for a long time (10 CPU hours or more on our 2.4 GHz reference machine) for the homopolymers of length 32 and 64, as shown in Table 3. We also investigated the scaling behaviour of REMC and BINMC with homopolymer length. We measured the median run-time to reach the global minimum over 20 runs for sequences of length 12, 24, and 32 amino acids (the sequence of length 64 was not used, since only BINMC reaches the lowest known energy). The resulting sets of three data points for each algorithm were each fitted with a line in a semi-logarithmic plot (which corresponds to fitting an exponential function to the original data in a way that counteracts over-fitting for large instance sizes). Based on this analysis, the median run-time required for finding (purportedly optimal) conformations appears to scale as 10^0.34·*N*-5.2 ^for REMC and as 10^0.28·*N*-4.7 ^for BINMC.

**Figure 6:**
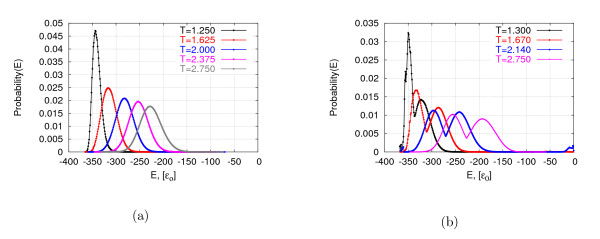
**Example of distributions of energies visited by different replicas in REMC and PHAT**. Distributions of energies visited by different replicas in a representative run of REMC (a) and of PHAT (b) for the homopolymer of length 64. The time cut-off used was 28 CPU min on our 2.4 GHz reference machine.

**Figure 7:**
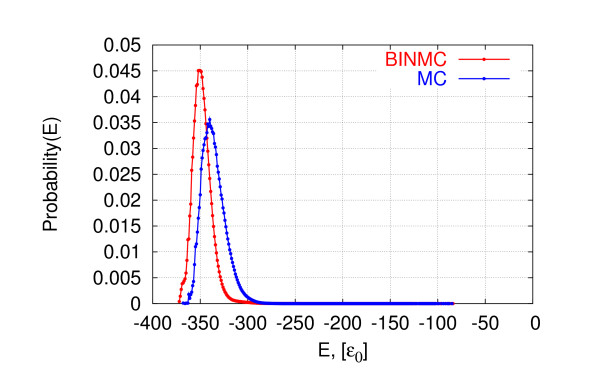
**Example of distributions of energies visited by BINMC**. Distributions of energies visited by MC and our new BINMC algorithm for the homopolymer of length 64. The time cut-off used was 28 CPU min on our 2.4 GHz reference machine.

Finally, we inspected the distribution of energies sampled by each method for the long homopolymer of length 64, based on approximately 5 × 10^9 ^conformations each. As seen in Figure 6, REMC and PHAT show typical energy distributions for each replica, as reported by Zhang and Skolnick [10]; as expected, in the case of PHAT, the probabilities of encountering low and high energies are elevated. Interesting differences are observed when examining the distribution of energies visited by MC and BINMC (see Figure 7): While our new algorithm samples energies according to the Boltzmann probability density function, the distributions are shifted towards lower energy values, reflecting the fact that BINMC tends to reach lower-energy conformations more efficiently.

## Discussion

Similar to the Model-based Search (MBS) method [28] introduced for off-lattice fragment insertion as used in the ROSETTA [15] algorithm, our bin framework stores promising candidate solutions for future reuse. However, unlike MBS, we developed and tested an adaptive diversification mechanism that varies based on the energy level considered and takes into account how different a conformation is from other conformations with the same energy. Additionally, the energy level of interest, which determines the highest energy conformations stored in the bin framework are allowed to have, and individual Hamming distance criteria used for each bin, are adapted according to the current estimate of the ground state energy. MBS does not have a mechanism comparable to our diversity criteria between stored conformations. In MBS, a number of elite conformations are stored, whose quality is measured using a score determined from their energies and the radius of the local minimum represented by them. (This radius is estimated from the distance to their nearest neighbours using root mean square deviation.) Another important distinction between BINMC and MBS is that in BINMC, the model (a pool of stored conformations) is updated during the search and influences the choice of a new candidate solution every *noImprRetrieve *steps, when search stagnation is detected. On the other hand, in MBS for the discrete off-lattice model with structural fragment insertion as described by Brunette and Brock [28], the choice of a new candidate solution is influenced at every step by the pool of conformations stored. Thus, MBS exploration of the search space is only dependent on the conformations stored [28]. Therefore, regions that are pruned based on the model are eliminated and not explored any further. In contrast, the bin framework provides only a subsidiary mechanism for generating candidate solutions when search stagnation is detected, and does not completely eliminate unexplored regions of the search space. This is achieved by running a non-model-based search (canonical MC) for a sufficiently long time to allow it to explore other regions of the search space.

The bin framework sorts conformations into bins representing different energy levels to make sure that the model contains as many energy levels of interest as possible while still reducing the search space. This aspect of the search is conceptually related to histogram-based sampling and search methods such as Multicannonical algorithm (MUCA) [24] and Energy Landscape Paving (ELP) method [21]. It should be noted, however, that our bin framework is a non-parametric model of the search space that consists of a diverse set of promising candidate conformations stored in the bins. MUCA, on the other hand, and to some degree ELP, are based on a parametric model of sampling all energy levels with the same probability without emphasis on low energies. In addition, our bin framework uses Hamming distance criteria that are based on the energy level of each bin to ensure that the respective sets of conformations stored are diverse and capture the overall funnel-like structure of the landscape.

Up to this point, we have focused on comparing BINMC with existing algorithms. We now turn our attention to the question how the performance of the BINMC algorithm depends on its parameters and the algorithm components controlled by them, and to the determination of good settings for these parameters. We conducted this investigation for the homopolymer of length 32, since reaching its best known energy level of -161 is challenging, but not too computationally expensive to preclude performing multiple successful runs for a large number of parameter settings. For each parameter configuration, we conducted 20 independent runs on our reference machine and recorded the time required in each of these to reach an energy of -161; from these runs, we then determined the average CPU time required to reach this target energy. To study the influence of the different parameters on algorithm performance, we varied one (or, in the case of closely related parameters, two) of them at a time, while keeping all other parameter values fixed. Unless indicated otherwise, the parameters kept fixed in these experiments were set to the following values: Δ*E *= 20 [*ε*_0_], Δ*E*_*i *_= 5 [*ε*_0_], *T*_*MC *_= 1.25 [*ε*_0_/*k*_*B*_], *T*_*bin *_= 4.344 [*ε*_0_/*k*_*B*_], *binCapacity *= 100, *HD*_*MAX *_= 0.6, *HD*_*MIN *_= 0.1, *noImprRetrieve *= 100 000 steps.

Here, we summarize the results of this study; details are provided in the supplementary material, see Additional file 1. The impact of the parameters on the performance of BINMC can be ranked as follows (in decreasing order):

1. the number of non-improving steps (over the best energy) that are performed before reaching into a bin and replacing the current conformation in the Monte Carlo run, *noImprRetrieve *(the optimal value seems to be around 1 000 000 steps which allows the underlying MC search to explore the current region of the landscape);

2. the ratio between the width of the energy of interest considered for binning, Δ*E*, and the bin temperature, *T*_*bin *_(the ratio that results in at least 0.01 probability of retrieval of the highest energy conformations works well);

3. the diversity criteria used during binning high- and low-energy conformations (the Hamming distance limits), values of *HD*_*min *_= 0.01 and *HD*_*max *_= 0.06 guarantee that we are selective enough when storing promising conformations at low and high energy levels correspondingly and result in the best performance;

4. the width of the energy window considered by each bin (*ΔE*_*i *_= 5 seem to provide optimal discretization of the search space); and

5. the capacity of bins, *cap*_*i *_(storing 100 conformations in each bin seems to work best, this provides required diversification during the search).

Furthermore, we made the following observations regarding parameter settings of our algorithm for the longer homopolymer of length 64:

1. a higher value of *noImprRetrieve *should be used (2 000 000 was found to work well), which indicates that longer search times are required to effectively explore the neighbourhood of the current conformation before reaching into the bin framework and replacing it with another promising conformation;

2. Δ*E *should be increased, which is consistent with the common belief that the barrier heights for longer homopolymers are higher, and *T*_*bin *_should be adjusted such that the ratio Δ*E/T*_*bin *_remains the same as for length 32;

3. Δ*E*_*i *_should be increased to *E*_*i *_= 10, which suggests that a coarser search space discretization may be beneficial for longer homopolymers; note that the combination of increases in Δ*E *and Δ*Ei *results in only a slight increase in the total number of bins, *numBins*;

4. the same values for *HD*_*min*_, *HD*_*max *_and *cap*_*i *_can be used as in the case of length 32.

Finally, our empirical results presented in Additional file 1 show that the BINMC algorithm performs substantially better than the simple restart strategy and than the pure Monte Carlo on which it is based. We also determined that all components of our algorithm are important for its efficiency, see Additional file 1. For example, the average time required for finding purportedly optimal conformations increases significantly (at least threefold for the homopolymer of length 32) when increasing *noImprRetrieve *until bin framework retrieval is performed very infrequently, when decreasing Δ*E *or *cap*_*i *_or when increasing the diversity thresholds *HD*_*min *_and *HD*_*max*_, resulting in very few conformations being stored, or when reducing the number of bins (by varying Δ*E*_*i *_for fixed Δ*E*).

## Conclusion

The bin framework introduced in this work is a general approach that can be used to augment existing conformation search methods in order to increase their ability to focus on promising regions of the search phase (intensification) and to effectively overcome stagnation in regions of sub-optimal conformations (diversification). As shown by our computational experiments, even very simple instantiations of the general bin framework can result in highly effective search algorithms.

In particular, our novel Bin Framework Monte Carlo algorithm (a combination of the bin framework and a simple Monte Carlo search procedure) surpasses Replica Exchange Monte Carlo search and its heuristic variant, Parallel-hat Tempering, in its ability to find (purportedly) minimum energy conformations for *β*-sheet homopolymers on the FCC lattice. Furthermore, using our new algorithm we have improved the best known solution for the homopolymer of length 64 from -387 to -391.

In future work, we plan to consider more advanced adaptive bin framework strategies that control search diversification and intensification reactively during the search, based on observed features of the search landscape. Additionally, we are planning to generalize our bin framework to work on partial as well as complete conformations, producing an efficient generalized framework that combines two distinct search strategies. Finally, we would like to extend our bin framework to address other models of protein structure, such as the FCC model with a more complex energy function or other discrete and continuous off-lattice models.

Given the results for *β*-sheet homopolymers on the FCC lattice achieved in this work, we believe that further investigation of our adaptive bin framework and its application to other protein structure prediction problems holds much promise.

## Methods

In this section, we provide a detailed description of the FCC lattice model, *β*-sheet energy potential, and experimental analysis performed to compare our approach to the existing methods from the literature.

### Face-Centered Cubic lattice model

In the FCC model, the polypeptide is restricted to a face-centered cubic lattice [11] that has 12 base set vectors:

*v*_*base *_= {**e**_1_, **e**_2_, ..., **e**_12_},

where **e**_1 _= (1, 1, 0), **e**_2 _= (1, -1, 0), **e**_3 _= (1, 0, 1), **e**_4 _= (1, 0, -1), **e**_5 _= (0, 1, 1), **e**_6 _= (0, 1, -1), **e**_7 _= (0, -1, 1), **e**_8 _= (0, -1, -1), **e**_9 _= (-1, 0, 1), **e**_10 _= (-1, 0, -1), **e**_11 _= (-1, 1, 0), **e**_12 _= (-1, -1, 0).

A protein chain of *N *residues is described by *N *- 1 vectors, where vector **v**_*i *_connects residues *i *and (*i *+ 1). The 12 base vectors allow for the following valence angles between each pair of vectors: 60°, 90°, 120°, and 180° [9].

### Beta sheet energy potential used with the FCC lattice

To model *β*-sheet proteins and the stiffness of the polymer chain, the following definition of an extended, *β *-type chain conformation was defined by Gront *et al*. [9]: three vectors are in an extended state if

1. the angles between vectors **v**_*i*-1 _and **v**_*i *_and between **v**_*i *_and **v**_*i*+1 _are greater than 90 degrees;

2. the dot product **v**_*i*-1_·**v**_*i*+1 _is larger than 0, which means that the angle between vectors **v**_*i*-1 _and **v**_*i*+1 _is less than 90 degrees.

The energy potential for this model is composed of two terms: the short-range potential *U*_*i*-1, *i*, *i*+1 _that depends on the three consecutive vectors in the chain (**v**_*i*-1_, **v**_*i*_, **v**_*i*+1_) and mimics conformational propensity to form an extended set of *β*-strands:

Ui−1,i,i+1={−εBif the triple vi−1,vi,vi+1 is in extended state,0,otherwise
 MathType@MTEF@5@5@+=feaafiart1ev1aaatCvAUfKttLearuWrP9MDH5MBPbIqV92AaeXatLxBI9gBaebbnrfifHhDYfgasaacH8akY=wiFfYdH8Gipec8Eeeu0xXdbba9frFj0=OqFfea0dXdd9vqai=hGuQ8kuc9pgc9s8qqaq=dirpe0xb9q8qiLsFr0=vr0=vr0dc8meaabaqaciaacaGaaeqabaqabeGadaaakeaacqWGvbqvdaWgaaWcbaGaemyAaKMaeyOeI0IaeGymaeJaeiilaWIaemyAaKMaeiilaWIaemyAaKMaey4kaSIaeGymaedabeaakiabg2da9maaceqabaqbaeaabiGaaaqaaiabgkHiTGGaciab=v7aLnaaBaaaleaacqWGcbGqaeqaaaGcbaGaeeyAaKMaeeOzayMaeeiiaaIaeeiDaqNaeeiAaGMaeeyzauMaeeiiaaIaeeiDaqNaeeOCaiNaeeyAaKMaeeiCaaNaeeiBaWMaeeyzauMaeeiiaaccbeGae4NDay3aaSbaaSqaaiabdMgaPjabgkHiTiabigdaXaqabaGccqGGSaalcqGF2bGDdaWgaaWcbaGaemyAaKgabeaakiabcYcaSiab+zha2naaBaaaleaacqWGPbqAcqGHRaWkcqaIXaqmaeqaaOGaeeiiaaIaeeyAaKMaee4CamNaeeiiaaIaeeyAaKMaeeOBa4MaeeiiaaIaeeyzauMaeeiEaGNaeeiDaqNaeeyzauMaeeOBa4MaeeizaqMaeeyzauMaeeizaqMaeeiiaaIaee4CamNaeeiDaqNaeeyyaeMaeeiDaqNaeeyzauMaeiilaWcabaGaeGimaaJaeiilaWcabaGaee4Ba8MaeeiDaqNaeeiAaGMaeeyzauMaeeOCaiNaee4DaCNaeeyAaKMaee4CamNaeeyzaugaaaGaay5Eaaaaaa@86FF@

and the long-range potential *V*_*i*, *j *_for two non-bonded chain residues, defined as:

Vi,j={+∞,for ri,j=0 and i≠j,−εA,for ri,j=1 (in lattice units) and |i−j|>1,0,for ri,j>1 (in lattice units)
 MathType@MTEF@5@5@+=feaafiart1ev1aaatCvAUfKttLearuWrP9MDH5MBPbIqV92AaeXatLxBI9gBaebbnrfifHhDYfgasaacH8akY=wiFfYdH8Gipec8Eeeu0xXdbba9frFj0=OqFfea0dXdd9vqai=hGuQ8kuc9pgc9s8qqaq=dirpe0xb9q8qiLsFr0=vr0=vr0dc8meaabaqaciaacaGaaeqabaqabeGadaaakeaacqWGwbGvdaWgaaWcbaGaemyAaKMaeiilaWIaemOAaOgabeaakiabg2da9maaceqabaqbaeaabmGaaaqaaiabgUcaRiabg6HiLkabcYcaSaqaaiabbAgaMjabb+gaVjabbkhaYjabbccaGiabdkhaYnaaBaaaleaacqWGPbqAcqGGSaalcqWGQbGAaeqaaOGaeyypa0JaeGimaaJaeeiiaaIaeeyyaeMaeeOBa4MaeeizaqMaeeiiaaIaemyAaKMaeyiyIKRaemOAaOMaeiilaWcabaGaeyOeI0ccciGae8xTdu2aaSbaaSqaaiabdgeabbqabaGccqGGSaalaeaacqqGMbGzcqqGVbWBcqqGYbGCcqqGGaaicqWGYbGCdaWgaaWcbaGaemyAaKMaeiilaWIaemOAaOgabeaakiabg2da9iabigdaXiabbccaGiabcIcaOiabbMgaPjabb6gaUjabbccaGiabbYgaSjabbggaHjabbsha0jabbsha0jabbMgaPjabbogaJjabbwgaLjabbccaGiabbwha1jabb6gaUjabbMgaPjabbsha0jabbohaZjabcMcaPiabbccaGiabbggaHjabb6gaUjabbsgaKjabbccaGiabcYha8jabdMgaPjabgkHiTiabdQgaQjabcYha8jabg6da+iabigdaXiabcYcaSaqaaiabicdaWiabcYcaSaqaaiabbAgaMjabb+gaVjabbkhaYjabbccaGiabdkhaYnaaBaaaleaacqWGPbqAcqGGSaalcqWGQbGAaeqaaOGaeyOpa4JaeGymaeJaeeiiaaIaeiikaGIaeeyAaKMaeeOBa4MaeeiiaaIaeeiBaWMaeeyyaeMaeeiDaqNaeeiDaqNaeeyAaKMaee4yamMaeeyzauMaeeiiaaIaeeyDauNaeeOBa4MaeeyAaKMaeeiDaqNaee4CamNaeiykaKcaaaGaay5Eaaaaaa@AA70@

where *r*_*i*, *j *_is the lattice distance between residues *i *and *j*.

For a chain of length *N*, the total energy is defined as:

E=∑i=2N−1Ui−1,i,i+1+∑i=1N∑j=1NVi,j(1−δij),
 MathType@MTEF@5@5@+=feaafiart1ev1aaatCvAUfKttLearuWrP9MDH5MBPbIqV92AaeXatLxBI9gBaebbnrfifHhDYfgasaacH8akY=wiFfYdH8Gipec8Eeeu0xXdbba9frFj0=OqFfea0dXdd9vqai=hGuQ8kuc9pgc9s8qqaq=dirpe0xb9q8qiLsFr0=vr0=vr0dc8meaabaqaciaacaGaaeqabaqabeGadaaakeaacqWGfbqrcqGH9aqpdaaeWbqaaiabdwfavnaaBaaaleaacqWGPbqAcqGHsislcqaIXaqmcqGGSaalcqWGPbqAcqGGSaalcqWGPbqAcqGHRaWkcqaIXaqmaeqaaOGaey4kaSYaaabCaeaadaaeWbqaaiabdAfawnaaBaaaleaacqWGPbqAcqGGSaalcqWGQbGAaeqaaOGaeiikaGIaeGymaeJaeyOeI0ccciGae8hTdq2aaSbaaSqaaiabdMgaPjabdQgaQbqabaGccqGGPaqkcqGGSaalaSqaaiabdQgaQjabg2da9iabigdaXaqaaiabd6eaobqdcqGHris5aaWcbaGaemyAaKMaeyypa0JaeGymaedabaGaemOta4eaniabggHiLdaaleaacqWGPbqAcqGH9aqpcqaIYaGmaeaacqWGobGtcqGHsislcqaIXaqma0GaeyyeIuoaaaa@5EA5@

where *δ*_*ij *_is the Kronecker delta (*δ*_*ij *_= 1 when *i *= *j*, and 0 otherwise) and the values of the force field parameters are defined as *ε*_*A *_:= 1.0 [*ε*_0_] and *ε*_*B *_:= 4.0 [*ε*_0_] (with *ε*_0 _:= 1.0 representing one unit of interaction energy) to model a semi-flexible polymer [9].

### Hamming distance criteria for the FCC lattice

For the FCC *β*-sheet energy potential the normalized average Hamming distance, *HD*, between the *β*-sheet energy sequence of a newly considered conformation *c *and all *β*-sheet energy sequences for the set *C' *of all conformations with the same energy that are already in the bin is calculated as follows.

HD=(∑k=1M∑i=2N−2Ui(c′k)/M−∑i=2N−2Ui(c))/(εB⋅(N−3)),
 MathType@MTEF@5@5@+=feaafiart1ev1aaatCvAUfKttLearuWrP9MDH5MBPbIqV92AaeXatLxBI9gBaebbnrfifHhDYfgasaacH8akY=wiFfYdH8Gipec8Eeeu0xXdbba9frFj0=OqFfea0dXdd9vqai=hGuQ8kuc9pgc9s8qqaq=dirpe0xb9q8qiLsFr0=vr0=vr0dc8meaabaqaciaacaGaaeqabaqabeGadaaakeaacqWGibascqWGebarcqGH9aqpdaqadaqaamaaqahabaWaaabCaeaacqWGvbqvdaWgaaWcbaGaemyAaKgabeaakiabcIcaOiqbdogaJzaafaWaaSbaaSqaaiabdUgaRbqabaGccqGGPaqkcqGGVaWlcqWGnbqtaSqaaiabdMgaPjabg2da9iabikdaYaqaaiabd6eaojabgkHiTiabikdaYaqdcqGHris5aOGaeyOeI0YaaabCaeaacqWGvbqvdaWgaaWcbaGaemyAaKgabeaakiabcIcaOiabdogaJjabcMcaPaWcbaGaemyAaKMaeyypa0JaeGOmaidabaGaemOta4KaeyOeI0IaeGOmaidaniabggHiLdaaleaacqWGRbWAcqGH9aqpcqaIXaqmaeaacqWGnbqta0GaeyyeIuoaaOGaayjkaiaawMcaaiabc+caViabcIcaOGGaciab=v7aLnaaBaaaleaacqWGcbGqaeqaaOGaeyyXICTaeiikaGIaemOta4KaeyOeI0IaeG4mamJaeiykaKIaeiykaKIaeiilaWcaaa@66F1@

where C={c′1,...,c′M}
 MathType@MTEF@5@5@+=feaafiart1ev1aaatCvAUfKttLearuWrP9MDH5MBPbIqV92AaeXatLxBI9gBaebbnrfifHhDYfgasaacH8akY=wiFfYdH8Gipec8Eeeu0xXdbba9frFj0=OqFfea0dXdd9vqai=hGuQ8kuc9pgc9s8qqaq=dirpe0xb9q8qiLsFr0=vr0=vr0dc8meaabaqaciaacaGaaeqabaqabeGadaaakeaacqWGdbWqcqGH9aqpcqGG7bWEcuWGJbWygaqbamaaBaaaleaacqaIXaqmaeqaaOGaeiilaWIaeiOla4IaeiOla4IaeiOla4IaeiilaWIafm4yamMbauaadaWgaaWcbaGaemyta0eabeaakiabc2ha9baa@3B62@, *U*_*i*_(*x*) denotes the *U*_*i*-1, *i*, *i*+1 _value for conformation *x*, and *ε*_*B *_= 4.0 [*ε*_0_] represents the energy contribution of each *β*-residue [9]. The inner sum ranges from *i *= 2 to *i *= *N *- 2, since the first residue and the two last residues can never be in the extended *β*-state.

### Empirical analysis

BINMC has been implemented in C++ and compiled using gcc (version 3.3.3) for the Linux operating system. The same holds for our implementations of three published algorithms for the same protein models: simple Monte Carlo (MC), Replica Exchange Monte Carlo (REMC) and Parallel-hat (PHAT) Monte Carlo search [9,10]; we had to re-implement these because the respective codes could not be obtained from the authors. All experiments were performed on PCs with 2.4 GHz Pentium IV CPUs, 256 Kb cache, and 1 Mb RAM, running Redhat Linux (our reference machine). Their run-time was measured in terms of the CPU time required to reach (or exceed) a specified energy level.

For our performance analysis we used the FCC *β*-homopolymers of length 12, 24, 36, and 64. The algorithms were evaluated based on a number of independent runs on each homopolymer. In most experiments, each run was terminated after a fixed CPU time limit (cut-off time) had been reached. From the distribution of energy levels over 10 independent runs, we determined the average energy, median energy, 25- and 75-percentiles as well as the lowest energy reached. To further evaluate the performance of our BINMC algorithm and the methods known from the literature, we followed the methodology of Hoos and Stützle [29] and analyzed run-time distributions (RTDs) of the algorithms, i.e., the (empirical) probability distributions over the run-time required to reach the lowest known (or, in some cases, certain sub-optimal) energy level for the respective homopolymer based on 100 independent runs.

## Authors' contributions

Both authors contributed to the development of ideas, design of experiments, analysis and interpretation of results, and the writing of the paper. AS implemented the proposed method and performed the computational experiments.

## Supplementary Material

Additional file 1 Additional data for an adaptive bin framework search method for a beta-sheet protein homopolymer model. In this supplementary file, we show additional low-energy conformations for the 64 amino acid homopolymer found by BINMC, provide details on experiments conducted to determine good parameter settings for BINMC and supply the necessary information to reconstruct the lowest energy conformations of homopolymers of length 12, 24, 32, and 64 found by our new algorithm. Click here for file

## References

[B1] Notling B (1993). Protein Folding Kinetics.

[B2] Ngo JT, Marks J, Karplus M (1992). Computational Complexity: Protein Structure Prediction and the Levinthal Paradox. Protein Engineering.

[B3] Unger R, Moult J (1993). Finding the Lowest Free Energy Conformation of a Protein is a NP-hard Problem: Proof and Implications. Bulletin of Mathematical Biology.

[B4] Hoos HH, Stützle T (2004). Stochastic Local Search: Foundations and Applications.

[B5] Bonneau R, Baker D (2001). Ab Initio Protein Structure Prediction: Progress and Prospects. Annual Review of Biophysics and Biomolecular Structure.

[B6] Kolinski A, Skolnick J (2004). Reduced Models of Proteins and their Applications. Polymer.

[B7] Park BH, Levitt M (1995). The Complexity and Accuracy of Discrete State Models of Protein Structure. Journal of Molecular Biology.

[B8] Bagci Z, Jernigan RL, Bahar I (1994). The Reactive Tabu Search. ORSA Journal on Computing.

[B9] Gront D, Kolinski A, Skolnick J (2000). Comparison of Three Monte Carlo Conformational Search Strategies for a Protein-like Homopolymer Model: Folding Thermodynamics and Identification of Low-Energy Structures. Journal of Chemical Physics.

[B10] Zhang Y, Skolnick J (2001). Parallel-Hat Tempering: A Monte Carlo Search Scheme for the Identification of Low-Energy Structures. Journal of Chemical Physics.

[B11] Pokarowski P, Kolinski A, Skolnick J (2003). A Minimal Physically Realistic Protein-Like Lattice Model: Designing and Energy Landscape that Ensures All-Or-None Folding to a Unique Native State. Biophysical Journal.

[B12] Aloy P, Stark A, Hadley C, Russel RB (2003). Predictions without Templates: New Folds, Secondary Structure, and Contacts in CASP5. Proteins: Structure, Function, and Genetics.

[B13] Jones DT (2001). Predicting Novel Protein Folds by Using FRAGFOLD. Proteins: Structure, Function, and Genetics Suppl.

[B14] Ortiz AR, Kolinski A, Rotkiewicz P, Ilkowski B, Skolnick J (1999). Ab Initio Folding of Proteins Using Restrains Derived from Evolutionary Information. Proteins: Structure, Function, and Genetics Suppl.

[B15] Simons K, Kooperberg C, Huang E, Baker D (1997). Assembly of Protein Tertiary Structures from Fragments with Similar Local Sequences Using Simulated Annealing and Bayesian Scoring Function. Journal of Molecular Biology.

[B16] Skolnick J, Kolinski A, Kihara D, Betancourt M, Rotkiewicz P, Boniecki M (2001). Ab Initio Protein Structure Prediction via a Combination of Threading, Lattice Folding, Clustering, and Structure Refinement. Proteins: Structure, Function, and Genetics Suppl.

[B17] Zhang Y, Kihara D, Skolnick J (2002). Local Energy Landscape Flattening: Parallel Hyperbolic Monte Carlo Sampling of Protein Folding. Proteins: Structure, Function, and Genetics.

[B18] Bowie JU, Eisenberg D (1994). An Evolutionary Approach to Folding Small *α*-helical Proteins that Use Sequence Information and an Empirical Guiding Fitness Function. Proceedings of the National Academy of Sciences of the USA.

[B19] Dandekar T, Argos P (1994). Folding the Main Chain of Small Proteins with the Genetic Algorithm. Journal of Molecular Biology.

[B20] Pedersen JT, Moult J (1997). Protein Folding Simulations with Genetic Algorithms and a Detailed Molecular Description. Journal of Molecular Biology.

[B21] Hansmann UHE (1999). Protein Folding Simulations in a Deformed Energy Landscape. The European Physical Journal B.

[B22] Mitsutake A, Sugita Y, Okamoto Y (2001). Generalized-Ensemble Algorithms for Molecular Simulations of Biopolymers. Biopolymers (Peptide Science).

[B23] Sugita Y, Okamoto Y (2004). Replica-Exchange Multicanonical Algorithm and Multicanonical Replica-Exchange Method for Simulating Systems with Rough Energy Landscape. Condensed Materials Archive.

[B24] Okamoto Y (2004). Generalized-Ensemble Algorithms: Enhanced Sampling Techniques for Monte Carlo and Molecular Dynamic Simulations. Journal of Molecular Graphics and Modeling.

[B25] Battiti R, Tecchiolli G (1994). The Reactive Tabu Search. ORSA Journal on Computing.

[B26] Plotkin SS, Onuchic JN (2002). Understanding Protein Folding with Energy Landscape Theory Part I: Basic Concepts. Quarterly Reviews of Biophysics.

[B27] Wenzel W, Hamacher K (1999). Stochastic Tunneling Approach for Global Minimization of Complex Potential Energy Landscapes. Physical Review Letters.

[B28] Brunette TJ, Brock O (2005). Improving Protein Structure Prediction with Model-based Search. Bioinformatics.

[B29] Hoos HH, Stützle T (1998). On the Empirical Evaluation of Las Vegas Algorithms. Proceedings of the 14th Conference on Uncertainty in Artificial Intelligence.

