# Supplementary material for: An adaptive bin framework search method for a beta-sheet protein homopolymer model
Source: BMC Bioinformatics. 2007 Apr 24;8:136. doi: 10.1186/1471-2105-8-136 (PMC1894818; doi:10.1186/1471-2105-8-136)
Supplement: Additional file 1 — Additional data for an adaptive bin framework search method for a beta-sheet protein homopolymer model. In this supplementary file, we show additional low-energy conformations for the 64 amino acid homopolymer found by BINMC, provide details on experiments conducted to determine good parameter settings for BINMC and supply the necessary information to reconstruct the lowest energy conformations of homopolymers of length 12, 24, 32, and 64 found by our new algorithm. [file 1471-2105-8-136-S1.pdf]

Additional file 1: Additional data for an adaptive bin framework search method for a beta-sheet protein homopolymer model

Abstract

In this supplementary file, we show additional low-energy conformations for the 64 amino acid homopolymer found by BINMC, provide details on experiments conducted to determine good parameter settings for BINMC and supply the necessary information to reconstruct the lowest energy conformations of homopolymers of length $12, 24, 32$, and 64 found by our new algorithm.

1 Low-energy conformations for the 64 amino acid homopolymer

Figures 1 and 2 show low energy conformations with energies -387 and -389 for the 64 amino acid homopolymer. As mentioned in our paper, the lowest energy found for this homopolymer was -391.

2 Parameter settings for BINMC

In this section we describe details of our study of the parameters of the BINMC algorithm. In order to determine reasonable values for all parameters and study the impact of parameter settings on BINMC's performance, we performed 20 independent runs for various parameter settings on the polymer of length 32, and in each of these we measured the CPU time required to reach the lowest known energy level of -161. To study the influence of different parameters, we varied one parameter (or, for closely related parameters, sometimes two parameters) at a time, while all other parameters were kept constant. Unless indicated otherwise, parameters for the homopolymer of length 32 were fixed at the following values: $\Delta E = 20$, $\Delta E_i = 5$, $T_{MC} = 1.25$, $T_{bin} = 4.344$, $binCapacity = 100$, $HD_{MAX} = 0.6$, $HD_{MIN} = 0.1$, $noImprRetrieve = 100\,000$ steps. These particular parameter settings have been determined in preliminary, informal experiments (not reported here). For each parameter setting, we conducted 20 independent runs of BINMC on

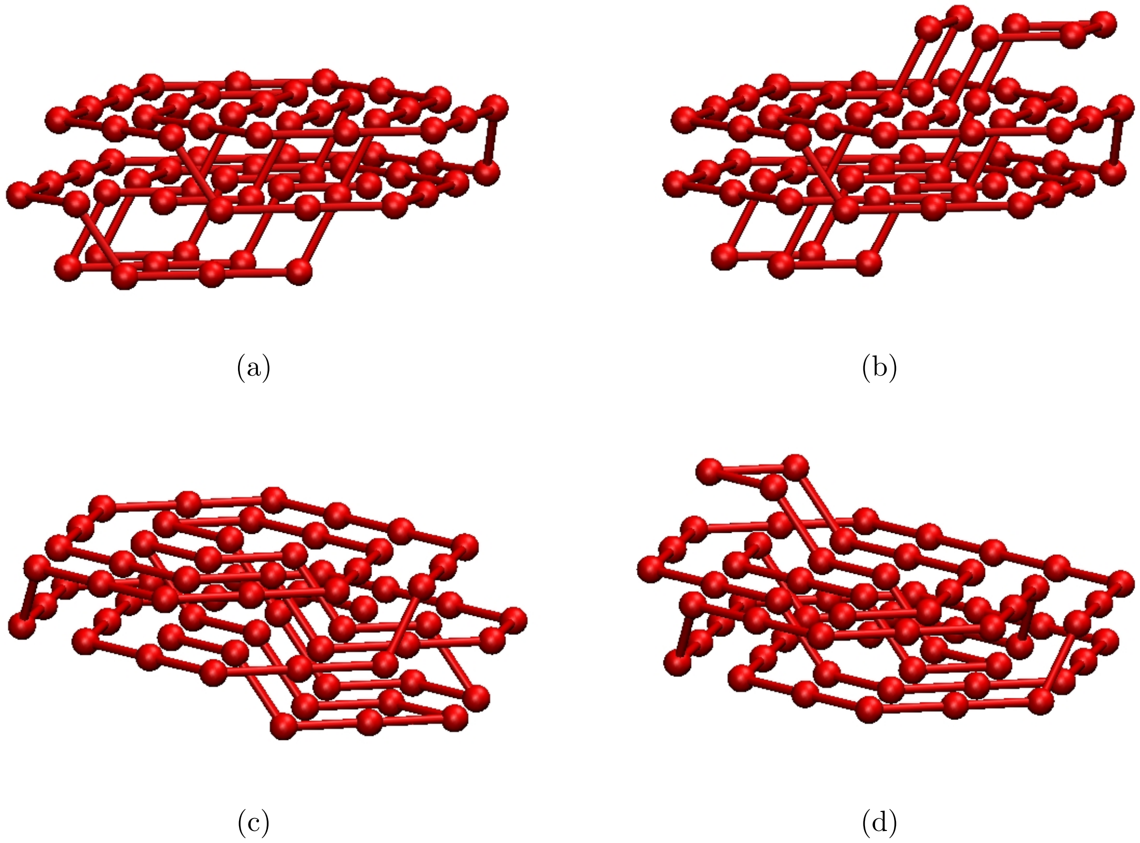

Fig. 1. Part (a) a low-energy conformation of the FCC 64 amino acid homopolymer found by BINMC (energy −387, short-range energy is −208, long-range energy is −179); part (b) another low-energy conformation of the FCC 64 amino acid homopolymer found in the same run of BINMC (energy −389, short-range energy is −212, long-range energy is −177). Part (c) a low-energy conformation of the FCC 64 amino acid homopolymer found by BINMC (energy −387, short-range energy is −208, long-range energy is −179); part (d) another low-energy conformation of the FCC 64 amino acid homopolymer found in the same run of BINMC (energy −389, short-range energy is −220, long-range energy is −169).

our 2.4 GHz reference machine and measured the average as well as the standard deviation of the CPU time required for reaching a conformation with energy −161.

2.1 The noImprRetrieve Parameter

The *noImprRetrieve* parameter controls the stringency of the stagnation criterion and hence the frequency of retrieving conformations from the bin system. To investigate the impact of this parameter on the performance of the BINMC algorithm, we conducted 20 independent runs for the homopolymer

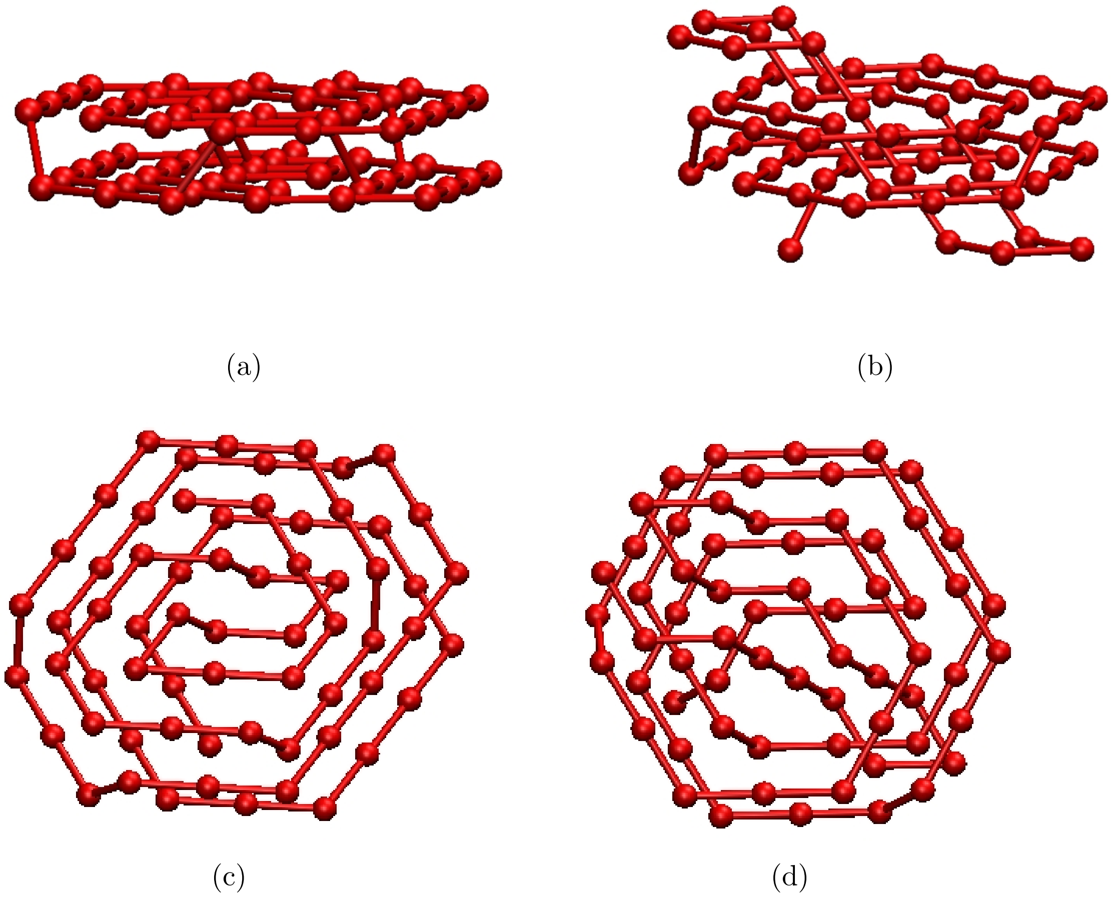

Fig. 2. Part (a) the low-energy conformation of the FCC 64 amino acid homopolymer found by our bin framework (energy −387, short-range energy is −220, long-range energy is −167); part (b) another low-energy conformation of the FCC 64 amino acid homopolymer found by our bin framework (energy −387, short-range energy is −220, long-range energy is −167). Part (c) the same conformation as in part (a), view from above; part (d) the same conformation as in part (b), view from above.

of length 32 with target energy −161 while varying *noImprRetrieve* from 1 000 to 2 000 000 steps. As can be seen from the results shown in Figure 3, the value of the *noImprRetrieve* parameter significantly affects performance of BINMC. For the homopolymer of length 32, a value close to 1 000 000 steps gives the best performance. For low values of *noImprRetrieve*, conformations are retrieved too frequently, resulting in a compromised ability of the algorithm to explore promising regions of the search landscape. On the other hand, for very large values of *noImprRetrieve*, BINMC's behaviour approaches that of a pure Monte Carlo search with temperature T_{MC}.

In informal experiments, we found evidence that the best value for the *noImprRetrieve* parameter increases with chain length; for the homopolymer of 64 amino acids *noImprRetrieve* = 2 000 000 steps results in good performance. This obser-

vation is consistent with the intuition that for longer chains, more time is required to explore promising local optima.

Additionally, we compared BINMC (which performs an adaptive retrieval of diverse conformations) with a simpler restart strategy: If there was no improvement on the best energy observed for $noImprRetrieve = 1\,000\,000$ steps, instead of retrieving a conformation from one of the bins we restarted the search with a newly constructed conformation. The resulting algorithm failed to reach the target energy of -161 within one week of CPU time on our 2.4 GHz reference machine (the lowest energy level reached was -159). Similarly, in the case of the homopolymer of length 64, the lowest energy level reached within one week of CPU time was -365.

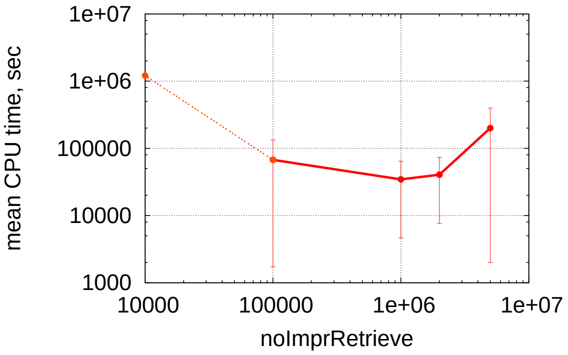

Fig. 3. Mean CPU time required for finding minimum energy conformations (-161) of the 32 amino acid homopolymer over 20 independent runs on our reference machine as a function of the number of non-improving steps (*noImprRetrieve*) before retrieving a conformation from bins. The error bars indicate the standard deviation over these runs. The dashed line indicates that energy of -161 was not reached after 2 weeks' CPU time on our reference machine.

2.2 The Energy Range ΔE and the Retrieval Temperature T_{bin}

The energy range ΔE determines the conformations that may be stored in the bin system, and the temperature T_{bin} controls the probability of retrieving conformations of various energies. Since it would be inefficient to store conformations that will later have a very low probability of being retrieved, the relative settings of these two parameters are important. The probability of retrieval of the conformation with the highest energy ($\hat{E} + \Delta E$) from the bin system is equal to $p = e^{-\beta_{bin}\Delta E}$, where $\beta_{bin} = 1/(k_B \cdot T_{bin})$. Thus, for example, if we would like to have at least a 1% chance of retrieving the highest energy conformation from the bin system ($e^{-\beta_{bin}\Delta E} \geq 0.01$), the following

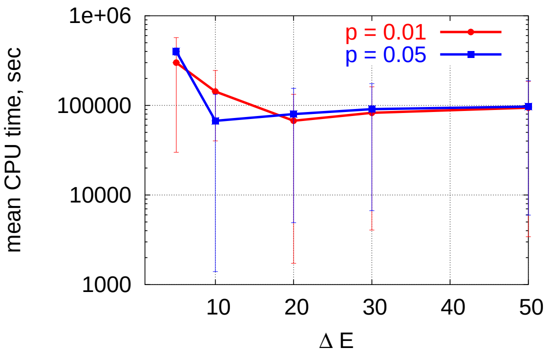

Fig. 4. Mean CPU time required for finding minimum energy conformations (-161) of the 32 amino acid homopolymer over 20 independent runs on our reference machine as a function of the energy range ΔE) and the retrieval temperature T_{bin} such that $e^{-\Delta E/T_{bin}} \geq p$. The error bars indicate the standard deviation over these runs.

condition must hold: $\frac{\Delta E}{T_{bin}} \geq 4.605$ (note that temperature is measured in units of $[\varepsilon_0/k_B]$). Therefore, if we fix a lower bound p on the probability of retrieval and one other parameter (either ΔE or T_{bin}, we chose ΔE), we can calculate the value of another parameter (T_{bin}). We fixed p at 0.01 and 0.05 and varied ΔE from 10 to $50[\varepsilon_0]$. Our results presented in Figure 4 indicate that BINMC shows a rather robust performance for various combinations of ΔE and T_{bin} settings. The performance deteriorates when the ΔE range is too small and also when we are only storing a few of the best conformations encountered. The search time increases slowly as ΔE becomes too large and when we are storing too many conformations. Each probability value (we tested $p = 0.01$ and $p = 0.05$) seems to have its optimal ΔE range, and as p increases, the optimal ΔE seems to decrease. This observation is consistent with the intuition that if the retrieval probability is increased, the search can perform well with a smaller energy range of interest, since the binned conformations have a higher chance of being retrieved. From experiments with the longer homopolymer of length 64 (not reported here) larger ΔE values result in good performance; particularly $\Delta E = 30$ and $T_{bin} = 6.522$, which implies $p \geq 0.01$, works well.

As for the relationship between T_{MC} and T_{bin}, here we only investigated the case when MC is run at a low temperature and the bin framework uses a high temperature for conformation retrieval. This scenario seems most promising, given the intuition that the basic search process has to be efficient in minimizing conformational energy, while the bin framework provides diversification and additional intensification at the same time. We found that running MC at high temperatures, for example, $T_{MC} = 2.0$ for both, the 32 and 64 amino acid polymers, does not result in very good performance. Therefore, T_{MC} was kept constant at 1.25, which is below the transition temperature of 1.8 $[\varepsilon_0/k_B]$

reported for the homopolymer of length 64, see Gront et al. (2000) and Zhang et al. (2001).

2.3 The Hamming Distance Limits HD_{MAX} and HD_{MIN}

The efficiency of our bin framework also depends on the diversity of conformations binned at each energy level. In this experiment, we investigated the role played by the Hamming distance criterion, which is controlled by the HD_{MAX} and HD_{MIN} parameters, for the overall performance of BINMC. First, we fixed HD_{MIN} (the fraction of residues that have to be different for low-energy conformations), and varied HD_{MAX} (the fraction of residues that have to be different for high-energy conformations) from 0.2 to 0.9. Note that setting any Hamming distance threshold to 0 would result in storing many copies of the same conformation. As seen in Figure 5, HD_{MAX} of about 0.6 seems to result in the best performance. As expected, if HD_{MAX} is set too low, the diversity of the set stored decreases and this impairs performance. If HD_{MAX} is set too high, very few conformations may exist to satisfy the corresponding stringent diversity criterion. Next, we fixed HD_{MAX}, and var-

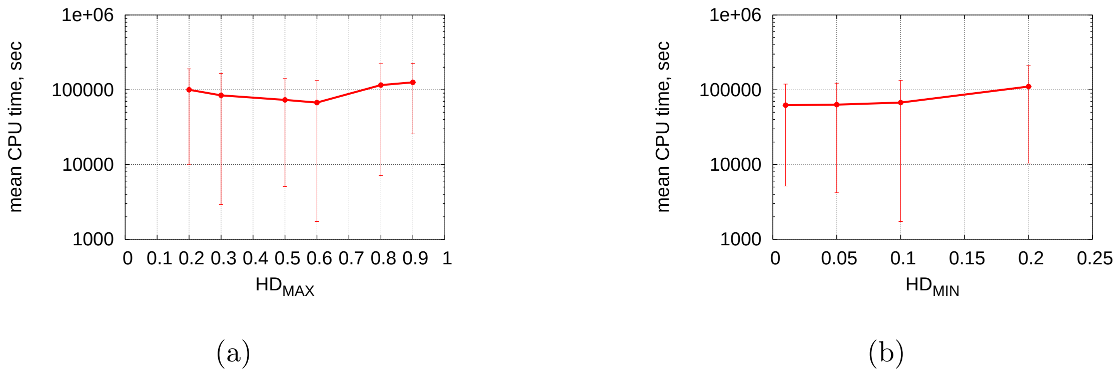

Fig. 5. Mean CPU time required for finding minimum energy conformations (−161) of the 32 amino acid homopolymer over 20 independent runs on our reference machine as a function of the Hamming distance limits; part (a): HD_{MAX} is varied, $HD_{MIN} = 0.1$ is kept constant; part(b): HD_{MIN} is varied, $HD_{MAX} = 0.6$ is kept constant. The error bars indicate the standard deviation over these runs.

ied HD_{MIN} from 0.01 to 0.9. As seen from Figure 5, if $HD_{MIN} > 0.1$ is set too high, the binning process becomes less efficient, since not all promising low-energy conformations are binned. This happens because there are fewer conformations at low-energy levels and these are more similar to each other than are higher-energy level conformations.

In informal experiments (not reported here), we found that similar values for

HD_{MAX} and HD_{MIN} appear to result in good performance for the homopolymer of length 64 (namely, $0.6 \leq HD_{MAX} \leq 0.8$ and $HD_{MIN} = 0.01$).

2.4 The Energy Window Width ΔE_i

In this experiment, we studied the performance of the BINMC algorithm a function of the energy window width, ΔE_i. Intuitively, ΔE_i controls the level of coarse-graining during the process of memorizing promising conformations. For simplicity, it is constant for all bins i in our implementation. Note that jointly, ΔE_i and the overall energy range ΔE determine the number of bins, $numBins$.

As seen in Figure 6, we varied ΔE_i from 1 (when every single energy level is stored in its own bin) to $10\varepsilon_0$ (when 10 different energy levels are combined in each bin). We observed that the performance of BINMC is quite robust with respect to the width of the energy window and that the optimal value appears to be around $5\varepsilon_0$. For the homopolymer of length 64, both $\Delta E_i = 5$ and $\Delta E_i = 10$ seem to result in good performance.

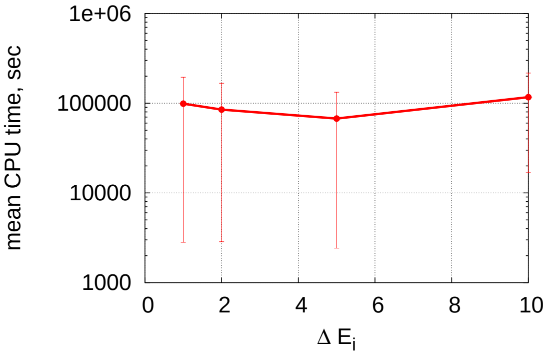

Fig. 6. Mean CPU time required for finding minimum energy conformations (−161) of the 32 amino acid homopolymer over 20 independent runs on our reference machine as a function of the energy window width ΔE_i. The error bars indicate the standard deviation over these runs.

2.5 The Bin Capacity cap_i

In our final experiment, we studied the impact of the bin capacity, which determines the number of conformations stored in each bin. As can be seen in Figure 7, good performance is generally obtained for $cap_i = 100$. Storing

too few or too many conformations results in a less efficient search process, because when too few conformations are stored the bin system does not provide effective search diversification, while storing too many conformations incurs unamortized computational overhead. For the homopolymer of length 64, a bin capacity of 100 appears to also work well.

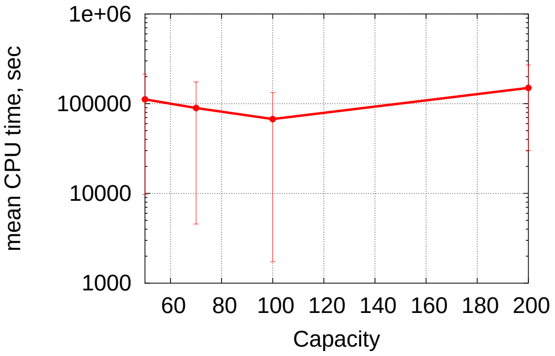

Fig. 7. Mean CPU time required for finding minimum energy conformations (−161) of the 32 amino acid homopolymer over 20 independent runs on our reference machine as a function of bin capacity. The error bars indicate the standard deviation over these runs.

3 Detailed description of lowest-energy conformations

A lowest energy conformation for the homopolymer of length 64 found by our BINMC is specified in detail in Tables 1-9; Tables 10-13 list an example of the best energy conformation for the homopolymer of length 32. Similarly, examples of the lowest energy conformations for the homopolymers of length 24 and 12 are provided in Tables 14-16, and 17-19, respectively.

$vector[\#]$	(x, y, z)
$\mathbf{v}[0]$	$(1, 1, 0)$
$\mathbf{v}[1]$	$(0, 1, -1)$
$\mathbf{v}[2]$	$(0, 1, -1)$
$\mathbf{v}[3]$	$(-1, 0, -1)$
$\mathbf{v}[4]$	$(-1, -1, 0)$
$\mathbf{v}[5]$	$(-1, -1, 0)$
$\mathbf{v}[6]$	$(0, -1, 1)$
$\mathbf{v}[7]$	$(0, -1, 1)$
$\mathbf{v}[8]$	$(1, 0, 1)$
$\mathbf{v}[9]$	$(1, 0, 1)$
$\mathbf{v}[10]$	$(1, 1, 0)$
$\mathbf{v}[11]$	$(1, 1, 0)$
$\mathbf{v}[12]$	$(0, 1, -1)$
$\mathbf{v}[13]$	$(0, 1, -1)$
$\mathbf{v}[14]$	$(0, 1, -1)$
$\mathbf{v}[15]$	$(-1, 0, -1)$
$\mathbf{v}[16]$	$(-1, 0, -1)$
$\mathbf{v}[17]$	$(-1, -1, 0)$
$\mathbf{v}[18]$	$(-1, -1, 0)$
$\mathbf{v}[19]$	$(-1, -1, 0)$
$\mathbf{v}[20]$	$(0, -1, 1)$
$\mathbf{v}[21]$	$(0, -1, 1)$
$\mathbf{v}[22]$	$(0, -1, 1)$
$\mathbf{v}[23]$	$(1, 0, 1)$
$\mathbf{v}[24]$	$(1, 0, 1)$
$\mathbf{v}[25]$	$(0, 1, 1)$
$\mathbf{v}[26]$	$(1, 1, 0)$
$\mathbf{v}[27]$	$(1, 1, 0)$
$\mathbf{v}[28]$	$(0, 1, -1)$
$\mathbf{v}[29]$	$(0, 1, -1)$
$\mathbf{v}[30]$	$(0, 1, -1)$
$\mathbf{v}[31]$	$(-1, 0, -1)$
$\mathbf{v}[32]$	$(-1, 0, -1)$
$\mathbf{v}[33]$	$(-1, -1, 0)$
$\mathbf{v}[34]$	$(-1, -1, 0)$
$\mathbf{v}[35]$	$(-1, -1, 0)$

Table 1
Vectors for the best found conformation of the 64 amino acid homopolymer (total energy $= -391$, short-range energy $= -212$, long-range energy $= -179$).

vector[#]	(x, y, z)
$\mathbf{v}[36]$	$(0, -1, 1)$
$\mathbf{v}[37]$	$(0, -1, 1)$
$\mathbf{v}[38]$	$(1, 0, 1)$
$\mathbf{v}[39]$	$(1, 0, 1)$
$\mathbf{v}[40]$	$(1, 1, 0)$
$\mathbf{v}[41]$	$(1, 1, 0)$
$\mathbf{v}[42]$	$(0, 1, -1)$
$\mathbf{v}[43]$	$(0, 1, -1)$
$\mathbf{v}[44]$	$(-1, 0, -1)$
$\mathbf{v}[45]$	$(-1, -1, 0)$
$\mathbf{v}[46]$	$(-1, -1, 0)$
$\mathbf{v}[47]$	$(0, -1, 1)$
$\mathbf{v}[48]$	$(1, 1, 0)$
$\mathbf{v}[49]$	$(1, 1, 0)$
$\mathbf{v}[50]$	$(1, 0, -1)$
$\mathbf{v}[51]$	$(1, 0, -1)$
$\mathbf{v}[52]$	$(1, 1, 0)$
$\mathbf{v}[53]$	$(0, -1, 1)$
$\mathbf{v}[54]$	$(-1, -1, 0)$
$\mathbf{v}[55]$	$(-1, 0, 1)$
$\mathbf{v}[56]$	$(-1, 0, 1)$
$\mathbf{v}[57]$	$(-1, -1, 0)$
$\mathbf{v}[58]$	$(1, 0, -1)$
$\mathbf{v}[59]$	$(0, 1, -1)$
$\mathbf{v}[60]$	$(1, 0, -1)$
$\mathbf{v}[61]$	$(0, 1, -1)$
$\mathbf{v}[62]$	$(1, 1, 0)$

Table 2
Continued, vectors for the best found conformation of the 64 amino acid homopolymer (total energy = -391, short-range energy = -212, long-range energy = -179).

triplet	θ_1	θ_2	θ_3	ε_{beta}
v_0, v_1, v_2	120	180	60	-4
v_1, v_2, v_3	180	120	60	-4
v_2, v_3, v_4	120	120	120	0
v_3, v_4, v_5	120	180	60	-4
v_4, v_5, v_6	180	120	60	-4
v_5, v_6, v_7	120	180	60	-4
v_6, v_7, v_8	180	120	60	-4
v_7, v_8, v_9	120	180	60	-4
v_8, v_9, v_{10}	180	120	60	-4
v_9, v_{10}, v_{11}	120	180	60	-4
v_{10}, v_{11}, v_{12}	180	120	60	-4
v_{11}, v_{12}, v_{13}	120	180	60	-4
v_{12}, v_{13}, v_{14}	180	180	0	-4
v_{13}, v_{14}, v_{15}	180	120	60	-4
v_{14}, v_{15}, v_{16}	120	180	60	-4
v_{15}, v_{16}, v_{17}	180	120	60	-4
v_{16}, v_{17}, v_{18}	120	180	60	-4
v_{17}, v_{18}, v_{19}	180	180	0	-4
v_{18}, v_{19}, v_{20}	180	120	60	-4
v_{19}, v_{20}, v_{21}	120	180	60	-4
v_{20}, v_{21}, v_{22}	180	180	0	-4
v_{21}, v_{22}, v_{23}	180	120	60	-4
v_{22}, v_{23}, v_{24}	120	180	60	-4
v_{23}, v_{24}, v_{25}	180	120	60	-4
v_{24}, v_{25}, v_{26}	120	120	60	-4
v_{25}, v_{26}, v_{27}	120	180	60	-4
v_{26}, v_{27}, v_{28}	180	120	60	-4
v_{27}, v_{28}, v_{29}	120	180	60	-4
v_{28}, v_{29}, v_{30}	180	180	0	-4
v_{29}, v_{30}, v_{31}	180	120	60	-4
v_{30}, v_{31}, v_{32}	120	180	60	-4
v_{31}, v_{32}, v_{33}	180	120	60	-4
v_{32}, v_{33}, v_{34}	120	180	60	-4
v_{33}, v_{34}, v_{35}	180	180	0	-4
v_{34}, v_{35}, v_{36}	180	120	60	-4
v_{35}, v_{36}, v_{37}	120	180	60	-4
v_{36}, v_{37}, v_{38}	180	120	60	-4
v_{37}, v_{38}, v_{39}	120	180	60	-4
v_{38}, v_{39}, v_{40}	180	120	60	-4
v_{39}, v_{40}, v_{41}	120	180	60	-4
v_{40}, v_{41}, v_{42}	180	120	60	-4

Table 3
Triplets of vectors, angles: θ_1 (between vectors $\mathbf{v_{i-1}}$ and $\mathbf{v_i}$), θ_2 (between vectors $\mathbf{v_i}$ and $\mathbf{v_{i+1}}$), θ_3 (between vectors $\mathbf{v_{i-1}}$ and $\mathbf{v_{i+1}}$), short-range energy contributions for the best found conformation of the 64 amino acid homopolymer (total energy $= -391$, short-range energy $= -212$, long-range energy $= -179$).

triplet	θ_1	θ_2	θ_3	ε_{beta}
v_{41}, v_{42}, v_{43}	120	180	60	-4
v_{42}, v_{43}, v_{44}	180	120	60	-4
v_{43}, v_{44}, v_{45}	120	120	120	0
v_{44}, v_{45}, v_{46}	120	180	60	-4
v_{45}, v_{46}, v_{47}	180	120	60	-4
v_{46}, v_{47}, v_{48}	120	60	180	0
v_{47}, v_{48}, v_{49}	60	180	120	0
v_{48}, v_{49}, v_{50}	180	120	60	-4
v_{49}, v_{50}, v_{51}	120	180	60	-4
v_{50}, v_{51}, v_{52}	180	120	60	-4
v_{51}, v_{52}, v_{53}	120	60	120	0
v_{52}, v_{53}, v_{54}	60	120	180	0
v_{53}, v_{54}, v_{55}	120	120	60	-4
v_{54}, v_{55}, v_{56}	120	180	60	-4
v_{55}, v_{56}, v_{57}	180	120	60	-4
v_{56}, v_{57}, v_{58}	120	60	180	0
v_{57}, v_{58}, v_{59}	60	120	120	0
v_{58}, v_{59}, v_{60}	120	120	0	-4
v_{59}, v_{60}, v_{61}	120	120	0	-4
v_{60}, v_{61}, v_{62}	120	120	60	-4

Table 4

Continued, triplets of vectors, angles: θ_1 (between vectors $\mathbf{v_{i-1}}$ and $\mathbf{v_i}$), θ_2 (between vectors $\mathbf{v_i}$ and $\mathbf{v_{i+1}}$), θ_3 (between vectors $\mathbf{v_{i-1}}$ and $\mathbf{v_{i+1}}$), short-range energy contributions for the best found conformation of the 64 amino acid homopolymer (total energy $= -391$, short-range energy $= -212$, long-range energy $= -179$).

#	Long-range interactions
1	10 interacts with 1
2	11 interacts with 1
3	12 interacts with 1
4	12 interacts with 2
5	13 interacts with 2
6	14 interacts with 2
7	14 interacts with 3
8	15 interacts with 3
9	15 interacts with 4
10	16 interacts with 4
11	17 interacts with 5
12	17 interacts with 4
13	18 interacts with 5
14	19 interacts with 6
15	19 interacts with 5
16	20 interacts with 7
17	20 interacts with 6
18	21 interacts with 7
19	22 interacts with 8
20	22 interacts with 7
21	23 interacts with 9
22	23 interacts with 8
23	24 interacts with 9
24	25 interacts with 9
25	25 interacts with 10
26	26 interacts with 10
27	26 interacts with 11
28	27 interacts with 11
29	28 interacts with 11
30	28 interacts with 12
31	29 interacts with 12
32	29 interacts with 13
33	30 interacts with 2
34	30 interacts with 13
35	30 interacts with 14
36	31 interacts with 3
37	31 interacts with 14
38	31 interacts with 15
39	32 interacts with 4
40	32 interacts with 15

Table 5
Long-range interactions for the best found conformation of the 64 amino acid homopolymer (total energy = −391, short-range energy = −212, long-range energy = −179).

#	Long-range interactions
41	32 interacts with 16
42	33 interacts with 5
43	33 interacts with 4
44	33 interacts with 17
45	34 interacts with 19
46	34 interacts with 5
47	34 interacts with 18
48	35 interacts with 20
49	35 interacts with 6
50	35 interacts with 19
51	36 interacts with 21
52	36 interacts with 7
53	36 interacts with 20
54	37 interacts with 22
55	37 interacts with 21
56	38 interacts with 23
57	38 interacts with 22
58	39 interacts with 24
59	39 interacts with 23
60	40 interacts with 24
61	40 interacts with 25
62	40 interacts with 9
63	41 interacts with 25
64	41 interacts with 26
65	41 interacts with 10
66	41 interacts with 27
67	42 interacts with 10
68	42 interacts with 27
69	42 interacts with 11
70	42 interacts with 1
71	42 interacts with 28
72	43 interacts with 1
73	43 interacts with 28
74	43 interacts with 12
75	43 interacts with 2
76	43 interacts with 29
77	43 interacts with 30
78	44 interacts with 2
79	44 interacts with 3
80	44 interacts with 30
81	44 interacts with 31
82	45 interacts with 3

Table 6
Continued, long-range interactions for the best found conformation of the 64 amino acid homopolymer (total energy = -391, short-range energy = -212, long-range energy = -179).

#	Long-range interactions
83	45 interacts with 33
84	45 interacts with 4
85	45 interacts with 31
86	45 interacts with 32
87	46 interacts with 35
88	46 interacts with 6
89	46 interacts with 34
90	46 interacts with 5
91	46 interacts with 33
92	47 interacts with 36
93	47 interacts with 7
94	47 interacts with 35
95	47 interacts with 6
96	48 interacts with 38
97	48 interacts with 37
98	48 interacts with 22
99	48 interacts with 8
100	48 interacts with 36
101	48 interacts with 7
102	49 interacts with 39
103	49 interacts with 38
104	49 interacts with 23
105	49 interacts with 40
106	49 interacts with 9
107	49 interacts with 8
108	50 interacts with 48
109	50 interacts with 8
110	50 interacts with 47
111	51 interacts with 47
112	51 interacts with 46
113	51 interacts with 44
114	51 interacts with 45
115	52 interacts with 6
116	52 interacts with 46
117	52 interacts with 5
118	52 interacts with 45
119	52 interacts with 3
120	52 interacts with 4
121	53 interacts with 5
122	53 interacts with 4

Table 7
Continued, long-range interactions for the best found conformation of the 64 amino acid homopolymer (total energy = -391, short-range energy = -212, long-range energy = -179).

#	Long-range interactions
123	54 interacts with 4
124	54 interacts with 17
125	54 interacts with 16
126	55 interacts with 53
127	55 interacts with 3
128	55 interacts with 4
129	55 interacts with 15
130	56 interacts with 52
131	56 interacts with 53
132	56 interacts with 3
133	57 interacts with 1
134	57 interacts with 51
135	57 interacts with 52
136	57 interacts with 44
137	57 interacts with 2
138	57 interacts with 3
139	58 interacts with 50
140	58 interacts with 42
141	58 interacts with 1
142	58 interacts with 51
143	58 interacts with 43
144	58 interacts with 44
145	59 interacts with 40
146	59 interacts with 49
147	59 interacts with 9
148	59 interacts with 41
149	59 interacts with 10
150	59 interacts with 50
151	59 interacts with 42
152	60 interacts with 9
153	60 interacts with 8
154	60 interacts with 10
155	60 interacts with 50
156	60 interacts with 58
157	60 interacts with 1
158	60 interacts with 57
159	61 interacts with 8
160	61 interacts with 7
161	61 interacts with 50
162	61 interacts with 47
163	61 interacts with 6
164	61 interacts with 51
165	61 interacts with 57

Table 8
Continued, long-range interactions for the best found conformation of the 64 amino acid homopolymer (total energy = -391, short-range energy = -212, long-range energy = -179).

#	Long-range interactions
166	61 interacts with 52
167	62 interacts with 6
168	62 interacts with 52
169	62 interacts with 56
170	62 interacts with 53
171	63 interacts with 6
172	63 interacts with 19
173	63 interacts with 5
174	63 interacts with 53
175	64 interacts with 5
176	64 interacts with 53
177	64 interacts with 18
178	64 interacts with 17
179	64 interacts with 54

Table 9
Continued, long-range interactions for the best found conformation of the 64 amino acid homopolymer (total energy = −391, short-range energy = −212, long-range energy = −179).

$vector[\#]$	(x, y, z)
$\mathbf{v}[0]$	$(1, 0, -1)$
$\mathbf{v}[1]$	$(1, -1, 0)$
$\mathbf{v}[2]$	$(1, -1, 0)$
$\mathbf{v}[3]$	$(0, -1, 1)$
$\mathbf{v}[4]$	$(-1, 0, 1)$
$\mathbf{v}[5]$	$(-1, 0, 1)$
$\mathbf{v}[6]$	$(-1, 1, 0)$
$\mathbf{v}[7]$	$(-1, 1, 0)$
$\mathbf{v}[8]$	$(0, 1, -1)$
$\mathbf{v}[9]$	$(0, 1, -1)$
$\mathbf{v}[10]$	$(1, 0, -1)$
$\mathbf{v}[11]$	$(1, 0, -1)$
$\mathbf{v}[12]$	$(1, -1, 0)$
$\mathbf{v}[13]$	$(1, -1, 0)$
$\mathbf{v}[14]$	$(1, -1, 0)$
$\mathbf{v}[15]$	$(0, -1, 1)$
$\mathbf{v}[16]$	$(0, -1, 1)$
$\mathbf{v}[17]$	$(-1, 0, 1)$
$\mathbf{v}[18]$	$(-1, 0, 1)$
$\mathbf{v}[19]$	$(0, 1, 1)$
$\mathbf{v}[20]$	$(-1, 1, 0)$
$\mathbf{v}[21]$	$(-1, 1, 0)$
$\mathbf{v}[22]$	$(0, 1, -1)$
$\mathbf{v}[23]$	$(0, 1, -1)$
$\mathbf{v}[24]$	$(1, 0, -1)$
$\mathbf{v}[25]$	$(1, 0, -1)$
$\mathbf{v}[26]$	$(1, -1, 0)$
$\mathbf{v}[27]$	$(1, -1, 0)$
$\mathbf{v}[28]$	$(0, -1, 1)$
$\mathbf{v}[29]$	$(0, -1, 1)$
$\mathbf{v}[30]$	$(-1, 0, 1)$

Table 10
Vectors for the best found conformation of the 32 amino acid homopolymer (total energy $= -161$, short-range energy $= -112$, long-range energy $= -49$).

triplet	θ_1	θ_2	θ_3	ε_{beta}
v_0, v_1, v_2	120	180	60	-4
v_1, v_2, v_3	180	120	60	-4
v_2, v_3, v_4	120	120	120	0
v_3, v_4, v_5	120	180	60	-4
v_4, v_5, v_6	180	120	60	-4
v_5, v_6, v_7	120	180	60	-4
v_6, v_7, v_8	180	120	60	-4
v_7, v_8, v_9	120	180	60	-4
v_8, v_9, v_{10}	180	120	60	-4
v_9, v_{10}, v_{11}	120	180	60	-4
v_{10}, v_{11}, v_{12}	180	120	60	-4
v_{11}, v_{12}, v_{13}	120	180	60	-4
v_{12}, v_{13}, v_{14}	180	180	0	-4
v_{13}, v_{14}, v_{15}	180	120	60	-4
v_{14}, v_{15}, v_{16}	120	180	60	-4
v_{15}, v_{16}, v_{17}	180	120	60	-4
v_{16}, v_{17}, v_{18}	120	180	60	-4
v_{17}, v_{18}, v_{19}	180	120	60	-4
v_{18}, v_{19}, v_{20}	120	120	60	-4
v_{19}, v_{20}, v_{21}	120	180	60	-4
v_{20}, v_{21}, v_{22}	180	120	60	-4
v_{21}, v_{22}, v_{23}	120	180	60	-4
v_{22}, v_{23}, v_{24}	180	120	60	-4
v_{23}, v_{24}, v_{25}	120	180	60	-4
v_{24}, v_{25}, v_{26}	180	120	60	-4
v_{25}, v_{26}, v_{27}	120	180	60	-4
v_{26}, v_{27}, v_{28}	180	120	60	-4
v_{27}, v_{28}, v_{29}	120	180	60	-4
v_{28}, v_{29}, v_{30}	180	120	60	-4

Table 11
Triplets of vectors, angles: θ_1 (between vectors $\mathbf{v_{i-1}}$ and $\mathbf{v_i}$), θ_2 (between vectors $\mathbf{v_i}$ and $\mathbf{v_{i+1}}$), θ_3 (between vectors $\mathbf{v_{i-1}}$ and $\mathbf{v_{i+1}}$), short-range energy contributions for the best found conformation of the 32 amino acid homopolymer (total energy $= -161$, short-range energy $= -112$, long-range energy $= -49$).

#	Long-range interactions
1	10 interacts with 1
2	11 interacts with 1
3	12 interacts with 1
4	12 interacts with 2
5	13 interacts with 2
6	14 interacts with 2
7	14 interacts with 3
8	15 interacts with 3
9	15 interacts with 4
10	16 interacts with 4
11	17 interacts with 5
12	17 interacts with 4
13	18 interacts with 5
14	19 interacts with 6
15	19 interacts with 5
16	20 interacts with 7
17	20 interacts with 6
18	21 interacts with 7
19	22 interacts with 8
20	22 interacts with 7
21	23 interacts with 9
22	23 interacts with 8
23	24 interacts with 9
24	24 interacts with 10
25	25 interacts with 10
26	25 interacts with 11
27	25 interacts with 1
28	26 interacts with 1
29	26 interacts with 12
30	26 interacts with 2
31	27 interacts with 2
32	27 interacts with 13
33	27 interacts with 14
34	28 interacts with 3
35	28 interacts with 14
36	28 interacts with 15
37	29 interacts with 4
38	29 interacts with 15
39	29 interacts with 16
40	30 interacts with 5

Table 12
Long-range interactions for the best found conformation of the 32 amino acid homopolymer (total energy = −161, short-range energy = −112, long-range energy = −49).

#	Long-range interactions
41	30 interacts with 4
42	30 interacts with 17
43	31 interacts with 19
44	31 interacts with 5
45	31 interacts with 18
46	32 interacts with 20
47	32 interacts with 6
48	32 interacts with 19
49	32 interacts with 21

Table 13
Continued, long-range interactions for the best found conformation of the 32 amino acid homopolymer (total energy = -161, short-range energy = -112, long-range energy = -49).

vector[#]	(x, y, z)
**v**[0]	$(1, 0, -1)$
**v**[1]	$(0, -1, -1)$
**v**[2]	$(0, -1, -1)$
**v**[3]	$(1, -1, 0)$
**v**[4]	$(0, -1, -1)$
**v**[5]	$(0, -1, -1)$
**v**[6]	$(-1, -1, 0)$
**v**[7]	$(-1, 0, -1)$
**v**[8]	$(0, 1, 1)$
**v**[9]	$(1, 0, 1)$
**v**[10]	$(0, 1, 1)$
**v**[11]	$(0, 1, 1)$
**v**[12]	$(0, 1, 1)$
**v**[13]	$(1, 1, 0)$
**v**[14]	$(0, 1, 1)$
**v**[15]	$(-1, -1, 0)$
**v**[16]	$(-1, 0, -1)$
**v**[17]	$(0, -1, -1)$
**v**[18]	$(0, -1, -1)$
**v**[19]	$(1, 0, -1)$
**v**[20]	$(0, -1, -1)$
**v**[21]	$(0, -1, -1)$
**v**[22]	$(1, -1, 0)$

Table 14
Vectors for the best found conformation of the 24 amino acid homopolymer (total energy = −109, short-range energy = −68, long-range energy = −41).

triplet	θ_1	θ_2	θ_3	ε_{beta}
v_0, v_1, v_2	120	180	60	-4
v_1, v_2, v_3	180	120	60	-4
v_2, v_3, v_4	120	120	0	-4
v_3, v_4, v_5	120	180	60	-4
v_4, v_5, v_6	180	120	60	-4
v_5, v_6, v_7	120	120	60	-4
v_6, v_7, v_8	120	60	120	0
v_7, v_8, v_9	60	120	180	0
v_8, v_9, v_{10}	120	120	0	-4
v_9, v_{10}, v_{11}	120	180	60	-4
v_{10}, v_{11}, v_{12}	180	180	0	-4
v_{11}, v_{12}, v_{13}	180	120	60	-4
v_{12}, v_{13}, v_{14}	120	120	0	-4
v_{13}, v_{14}, v_{15}	120	60	180	0
v_{14}, v_{15}, v_{16}	60	120	120	0
v_{15}, v_{16}, v_{17}	120	120	60	-4
v_{16}, v_{17}, v_{18}	120	180	60	-4
v_{17}, v_{18}, v_{19}	180	120	60	-4
v_{18}, v_{19}, v_{20}	120	120	0	-4
v_{19}, v_{20}, v_{21}	120	180	60	-4
v_{20}, v_{21}, v_{22}	180	120	60	-4

Table 15
Triplets of vectors, angles: θ_1 (between vectors $\mathbf{v_{i-1}}$ and $\mathbf{v_i}$), θ_2 (between vectors $\mathbf{v_i}$ and $\mathbf{v_{i+1}}$), θ_3 (between vectors $\mathbf{v_{i-1}}$ and $\mathbf{v_{i+1}}$), short-range energy contributions for the best found conformation of the 24 amino acid homopolymer (total energy $= -109$, short-range energy $= -68$, long-range energy $= -41$).

#	Long-range interactions
1	10 interacts with 8
2	11 interacts with 8
3	11 interacts with 7
4	11 interacts with 6
5	12 interacts with 6
6	12 interacts with 5
7	13 interacts with 4
8	13 interacts with 5
9	14 interacts with 3
10	15 interacts with 3
11	15 interacts with 2
12	16 interacts with 2
13	17 interacts with 14
14	17 interacts with 1
15	17 interacts with 2
16	17 interacts with 15
17	18 interacts with 14
18	18 interacts with 3
19	18 interacts with 1
20	18 interacts with 2
21	19 interacts with 13
22	19 interacts with 4
23	19 interacts with 14
24	19 interacts with 3
25	20 interacts with 12
26	20 interacts with 13
27	20 interacts with 4
28	21 interacts with 12
29	21 interacts with 6
30	21 interacts with 4
31	21 interacts with 5
32	22 interacts with 10
33	22 interacts with 11
34	22 interacts with 7
35	22 interacts with 6
36	23 interacts with 9
37	23 interacts with 10
38	23 interacts with 8
39	23 interacts with 7
40	24 interacts with 8
41	24 interacts with 7

Table 16
Long-range interactions for the best found conformation of the 24 amino acid homopolymer (total energy = -109, short-range energy = -68, long-range energy = -41).

$vector[\#]$	(x, y, z)
$\mathbf{v}[0]$	$(-1, -1, 0)$
$\mathbf{v}[1]$	$(0, -1, 1)$
$\mathbf{v}[2]$	$(0, -1, 1)$
$\mathbf{v}[3]$	$(1, -1, 0)$
$\mathbf{v}[4]$	$(1, -1, 0)$
$\mathbf{v}[5]$	$(1, 0, -1)$
$\mathbf{v}[6]$	$(-1, 1, 0)$
$\mathbf{v}[7]$	$(-1, 1, 0)$
$\mathbf{v}[8]$	$(0, 1, -1)$
$\mathbf{v}[9]$	$(0, 1, -1)$
$\mathbf{v}[10]$	$(-1, 1, 0)$

Table 17
Vectors for the best found conformation of the 12 amino acid homopolymer (total energy = −39, short-range energy = −28, long-range energy = −11).

$triplet$	θ_1	θ_2	θ_3	ε_{beta}
v_0, v_1, v_2	120	180	60	−4
v_1, v_2, v_3	180	120	60	−4
v_2, v_3, v_4	120	180	60	−4
v_3, v_4, v_5	180	120	60	−4
v_4, v_5, v_6	120	60	180	0
v_5, v_6, v_7	60	180	120	0
v_6, v_7, v_8	180	120	60	−4
v_7, v_8, v_9	120	180	60	−4
v_8, v_9, v_{10}	180	120	60	−4

Table 18
Triplets of vectors, angles: θ_1 (between vectors $\mathbf{v_{i-1}}$ and $\mathbf{v_i}$), θ_2 (between vectors $\mathbf{v_i}$ and $\mathbf{v_{i+1}}$), θ_3 (between vectors $\mathbf{v_{i-1}}$ and $\mathbf{v_{i+1}}$), short-range energy contributions for the best found conformation of the 12 amino acid homopolymer (total energy = −39, short-range energy = −28, long-range energy = −11).

#	Long-range interactions
1	8 interacts with 5
2	8 interacts with 6
3	9 interacts with 4
4	9 interacts with 5
5	9 interacts with 3
6	10 interacts with 3
7	10 interacts with 2
8	11 interacts with 2
9	11 interacts with 1
10	12 interacts with 2
11	12 interacts with 1

Table 19
Long-range interactions for the best found conformation of the 12 amino acid homopolymer (total energy = -39, short-range energy = -28, long-range energy = -11).
